# Association of the Patient Protection and Affordable Care Act With Ambulatory Quality, Patient Experience, Utilization, and Cost, 2014-2016

**DOI:** 10.1001/jamanetworkopen.2022.18167

**Published:** 2022-06-17

**Authors:** David M. Levine, Rohan Chalasani, Jeffrey A. Linder, Bruce E. Landon

**Affiliations:** 1 Division of General Internal Medicine and Primary Care, Brigham and Women’s Hospital, Boston, Massachusetts; 2 Harvard Medical School, Boston, Massachusetts; 3 Division of General Internal Medicine, Northwestern University Feinberg School of Medicine, Chicago, Illinois; 4 Department of Health Care Policy, Harvard Medical School, Boston, Massachusetts; 5 Division of General Medicine, Beth Israel Deaconess Medical Center, Boston, Massachusetts

## Abstract

**Importance:**

The Patient Protection and Affordable Care Act (ACA) expanded Medicaid eligibility at the discretion of states to US individuals earning up to 138% of the federal poverty level (FPL) and made private insurance subsidies available to most individuals earning up to 400% of the FPL. Its national impact remains debated.

**Objective:**

To determine the association of the ACA with ambulatory quality, patient experience, utilization, and cost.

**Design, Setting, and Participants:**

This cross-sectional study used difference-in-differences (DiD) analyses comparing outcomes before (2011-2013) and after (2014-2016) ACA implementation for US adults aged 18 to 64 years with income below and greater than or equal to 400% of the FPL. Participants were respondents to the Medical Expenditure Panel Survey, a nationally representative annual survey. Data analysis was performed from January 2021 to March 2022.

**Exposures:**

ACA implementation.

**Main Outcomes and Measures:**

For quality and experience, this study examined previously published composites based on individual measures, including high-value care composites (eg, preventive testing) and low-value care composites (eg, inappropriate imaging), an overall patient experience rating, a physician communication composite, and an access-to-care composite. For utilization, outpatient, emergency, and inpatient encounters and prescribed medicines were examined. Overall and out-of-pocket expenditures were analyzed for cost.

**Results:**

The total sample included 123 171 individuals (mean [SD] age, 39.9 [13.4] years; 65 034 women [52.8%]). After ACA implementation, adults with income less than 400% of the FPL received increased high-value care (diagnostic and preventive testing) compared with adults with income 400% or higher of the FPL (change from 70% to 72% vs change from 84% to 84%; adjusted DiD, 1.20%; 95% CI, 0.18% to 2.21%; *P* = .02) with no difference in any other quality composites. Individuals with income less than 400% of the FPL had larger improvements in experience, communication, and access composites compared with those with income greater than or equal to 400% of the FPL (global rating of health, change from 69% to 73% vs change from 79% to 81%; adjusted DiD, 2.12%; 95% CI, 0.18% to 4.05%; *P* = .03). There were no differences in utilization or cost, except that receipt of primary care increased for those with lower income vs those with higher income (change from 65% to 66% vs change from 80% to 77%; adjusted DiD, 2.97%; 95% CI, 1.18% to 4.77%; *P* = .001) and total out-of-pocket expenditures decreased for those with lower income vs those with higher income (change from $504 to $439 vs from $757 to $769; adjusted DiD, −$105.50; 95% CI, −$167.80 to −$43.20; *P* = .001).

**Conclusions and Relevance:**

In this cross-sectional national study, the ACA was associated with improved patient experience, communication, and access and decreased out-of-pocket expenditures, but little or no change in quality, utilization, and total cost.

## Key Points

**Question:**

How were ambulatory quality, patient experience, utilization, and cost associated with implementation of the Patient Protection and Affordable Care Act (ACA)?

**Findings:**

In this nationally representative cross-sectional study of 123 171 individuals, the ACA was associated with more high-value diagnostic and preventive testing, improved patient experience and access, and decreased out-of-pocket expenditures for lower income US individuals. The ACA was not associated with changes to most quality measures, utilization, or the total cost of care.

**Meaning:**

These findings suggest that policy makers and health system leaders seeking to further improve value should combine insurance expansion with additional policy initiatives to see broader improvements in care.

## Teaser

This cross-sectional study examines the association of the Patient Protection and Affordable Care Act (ACA) with ambulatory quality, patient experience, utilization, and cost.

## Introduction

The Patient Protection and Affordable Care Act (ACA) of 2010 expanded Medicaid eligibility at the discretion of states to individuals earning up to 138% of the federal poverty level (FPL). In addition, for private insurance purchased via the ACA insurance exchanges, the ACA made premium subsidies available to individuals earning between 100% and 400% of the FPL and cost-sharing subsidies for deductibles and copayments available to those earning less than 250% of the FPL.

Several studies have examined the shorter-term associations of the ACA in its first 2 years of implementation with access, cost, and utilization. Medicaid expansion under the ACA was associated with increased insurance coverage and reports of improved access to care.^1,2,3,4^ In addition, Medicaid expansion was associated with improved self-reported health and shifts in utilization away from emergency departments toward outpatient and preventive care,^5^ as well as improved affordability of care.^4^ More broadly, coverage expansion under the ACA was associated with less out-of-pocket spending in its first 2 years, especially among low-income individuals.^6^ Many of these findings extend to young adults^7,8^ and low-income and middle-income families with children.^9^ In the few studies that have examined these trends beyond the first 2 years of coverage expansion, increases in coverage, access, and affordability as well as shifts in utilization have persisted through the first 3 years of ACA implementation.^10,11^

The association of coverage expansion under the ACA with quality of care remains largely unexamined, although 2 initial studies^5,12^ have identified an association between Medicaid expansion and patient-reported access to and experiences with chronic care. In addition, among patients at federally funded health centers, Medicaid expansion was associated with improved quality in asthma treatment, Papanicolaou testing, body mass index assessment, and hypertension control, but not differences in other examined measures.^13^ Other studies have identified an association between Medicaid expansion and reduced mortality in 3 states,^14^ for patients with end-stage kidney disease,^15^ and for certain cancers due to earlier detection.^16,17,18,19^

A more complete and national understanding of the longer-term association between the implementation of the ACA and quality and experience of care in the US could better inform current and future policy decisions. Thus, in this cross-sectional study, we examined the association of the ACA with changes in high-value care, low-value care, patient experience, utilization, and cost using data from the nationally representative Medical Expenditure Panel Survey (MEPS). We hypothesized that, compared with individuals with income at or above 400% of the FPL, those with income below 400% of the FPL, who were the main targets of the ACA through both Medicaid expansion and the availability of subsidies to purchase private insurance in the exchanges, would have differential improvements associated with the ACA.

## Methods

### Overview

If the ACA was effective on a national scale, we would expect to observe greater improvements among those with income below 400% of the FPL than those with income at or above 400% of the FPL, because this was the principal population impacted by insurance expansion (including both Medicaid expansion and the availability of subsidies in the individual insurance market) under the ACA. We compared these 2 groups with a difference-in-differences (DiD) approach to account for secular trends and between-group differences before (pooling years 2011-2013) and after (pooling years 2014-2016) the ACA’s implementation. The Harvard Medical School institutional review board determined this study not to be human participants research; thus, informed consent was not needed in accordance with 45 CFR §46. We followed the Strengthening the Reporting of Observational Studies in Epidemiology (STROBE) reporting guidelines for cross-sectional studies.

### Data Source

We analyzed 2011 to 2016 data from the MEPS. We did not analyze currently available years 2017 and 2018 for 2 reasons: (1) changes to the ACA by the Trump Administration reduced the number of individuals with insurance^20^; and (2) the MEPS lacked key variables related to quality of care after 2016. The MEPS is a nationally representative annual survey of the noninstitutionalized US civilian population drawn from respondents to the National Health Interview Survey.^21^ The MEPS uses a complex survey design that delivers English or Spanish computer-assisted personal interviews to collect detailed data from individual respondents over a 2-year period on demographic characteristics (including race and ethnicity, which were analyzed in this study because both have been associated with Medicaid status), health conditions, health status, medical services utilization, medications, cost, source of payments, health insurance coverage, income, employment, experience with care, and access to care. From 2011 to 2016, annual response rates ranged from 46% to 56% (mean, 51%).

The MEPS supplements and validates self-reported information by contacting respondents’ clinicians (mean response rate, 87%), hospitals (85%), pharmacies (79%), and employers (73%). Clinicians specify details regarding office visits (eg, diagnosis, diagnostic tests, and cost), hospitals specify admissions, pharmacies specify medications, and employers specify insurance plan particulars.

The MEPS also includes 2 additional mail-back surveys: the adult self-administered questionnaire and the diabetes care survey. The self-administered questionnaire includes items from the Consumer Assessment of Healthcare Providers and Systems survey, the Short Form–12, and additional items measuring respondents’ attitudes about health care (annual response rate range, 88%-94%). The diabetes care survey, administered to respondents with self-reported diabetes, includes items related to diabetes care (annual response rate range, 84%-90%).

We restricted our analyses to the adult population aged 18 to 64 years. Sample sizes ranged from 23 697 to 26 509 respondents per year.

### Clinical Quality Measures

To define and measure outpatient quality, we developed clinical quality measures and quality composites from MEPS as previously described (eTable 1 in the Supplement).^22^ We evaluated performance on 35 clinical quality measures, including 25 high-value care measures and 10 low-value care measures. We excluded low-value cancer screening and inappropriate medication prescribing for older adults, as the population of interest was too young. From these measures, we constructed 5 clinically meaningful high-value (underuse) composites (eg, recommended cancer screening), where delivery of the service is likely of benefit to the respondent, and 3 low-value (overuse) composites (eg, avoidance of imaging in specific clinical situations), where delivery of the service is considered either inappropriate or of little or no benefit.

To calculate performance for each measure, we first identified those respondents who were eligible for the measure (eg, those with diabetes) and then whether they received the particular care (eg, retinal examination). To calculate composites, we divided all instances in which recommended care was delivered (for high-value measures) or avoided (for low-value measures) by the number of times respondents were eligible for care in the category, as others have done.^23^

### Respondent Experience, Communication, and Access Measures

We evaluated a global rating measure that asked about respondent experience with all health care practitioners (ranging from 0 [worst health care possible] to 10 [best health care possible]). We also evaluated a physician communication composite that asked 4 items (eg, “How often did the doctor spend enough time with you?”) and an access to care composite that included 2 items (eg, “How often did you get a medical appointment as soon as wanted?”).^22^ Responses were coded from never (1) to always (4). To better discriminate changes over time, we dichotomized (ie, top boxed) all measures such that a positive response included 8, 9, or 10 for the items scored from 0 to 10 and 4 for the items scored from 1 to 4, similar to Healthcare Effectiveness Data and Information Set analyses.^24^ We calculated both experience composites by first computing the mean for each respondent and then computing the mean across respondents.

### Utilization Measures

We evaluated utilization by characterizing yearly encounters (office and outpatient hospital visits, emergency department visits, and hospital admissions) and prescribed medicine fills. We also examined the percentage of respondents with an annual preventive visit and with primary care. The MEPS obtains these data first through self-report from respondents during each interview. To corroborate these reports, a follow-back survey collects data from a sample of medical practitioners and pharmacies used by respondents. Whenever conflicting data arise, the health professional data are retained over respondent data.

### Cost Measures

We evaluated the cost of care by characterizing total and out-of-pocket expenditures overall and in the office, hospital outpatient department, inpatient setting, and pharmacy. The MEPS calculates expenditures by asking respondents regarding all payments they made for each service. They aggregate this with payment data from payers (not including over-the-counter medications). Where data are missing, the MEPS uses an imputation process.^25^ MEPS cost data, although different in method from other nationally representative sources, have been used in multiple publications and government analyses.^26,27,28^

### Statistical Analysis

In all analyses, we generated national estimates as recommended by the MEPS by using survey estimation weights, primary sampling unit clusters, and sampling strata, which account for the complex survey design of the MEPS and nonresponse.^29,30^ To examine whether performance was improved at the end of the study period compared with the beginning, we compared pooled composites in 2011 to 2013 vs 2014 to 2016 using a DiD analysis, through multivariable linear regression with generalized least squares estimation and Taylor series linearization to account for the MEPS survey design, adjusting for all variables in Table 1 and whether respondents had primary care. To examine parallel trends in the pre-ACA period, we assessed the interaction of time with our income groups in 2011 to 2013. None of our quality composites, experience composites, or cost measures showed significant differences in trends in the pre-ACA period, except for 3 utilization measures (eTable 2 and eFigures 1, 2, 3, 4, 5, 6, and 7 in the Supplement). Observations with missing values were excluded from analyses, although the MEPS imputes values for missing expenditure data and several occupational and demographic variables and missing values were generally quite rare at the item level.^25^ To examine third-year associations, we compared 2011 to 2013 and 2016. This did not meaningfully alter our results and is presented in eTable 3 and eTable 4 in the Supplement.

**Table 1.** Characteristics of US Individuals Aged 19 to 64 Years Before (2011-2013) and After (2014-2016) the ACA, by Annual Household Income

Characteristic	Participants, % (95% CI)^a^			
	Annual household income <400% FPL		Annual household income ≥400% FPL	
	Before ACA (n = 45 811)	After ACA (n = 41 900)	Before ACA (n = 17 693)	After ACA (n = 17 767)
Age, mean (95% CI), y	39 (39-39)	39 (39-39)	44 (43-44)	43 (43-44)
Female sex	52 (52-53)	52 (52-53)	49 (48-49)	49 (48-49)
Race and ethnicity				
Hispanic	22 (20-24)	23 (21-26)	9 (8-10)	10 (9-11)
Non-Hispanic				
Asian	5 (4-6)	5 (4-6)	7 (6-8)	8 (7-9)
Black	15 (13-17)	16 (14-18)	8 (7-8)	8 (7-9)
White	56 (53-58)	53 (50-55)	75 (73-77)	71 (69-73)
Other or multiple^b^	2 (2-3)	3 (3-4)	2 (2-3)	3 (2-3)
Insurance				
Any private	56 (54-57)	58 (56-60)	92 (91-93)	93 (92-94)
Public only	18 (17-19)	24 (23-26)	1 (1-2)	2 (2-3)
Uninsured	26 (25-27)	18 (17-19)	7 (6-7)	5 (4-5)
US Census region				
Northeast	16 (15-18)	15 (14-16)	21 (19-23)	21 (19-24)
Midwest	21 (20-23)	21 (19-22)	21 (19-23)	21 (19-23)
South	39 (37-41)	40 (38-43)	34 (32-37)	33 (31-36)
West	24 (22-25)	24 (22-26)	23 (22-25)	24 (22-26)
Partner status				
Married or partnered	43 (42-44)	42 (41-43)	66 (65-67)	65 (64-66)
Widowed	2 (2-2)	2 (2-2)	1 (1-1)	1 (1-1)
Divorced or separated	17 (16-17)	16 (15-16)	9 (9-10)	9 (8-9)
Never married	38 (37-39)	40 (39-42)	24 (23-25)	26 (25-27)
Education				
Less than high school	19 (18-20)	20 (19-21)	5 (5-6)	5 (4-5)
High school, general educational development, or some college	64 (63-65)	63 (62-64)	46 (44-48)	48 (46-49)
Bachelor’s degree	13 (12-14)	13 (12-13)	30 (28-31)	28 (27-30)
More than bachelor’s degree	4 (4-5)	4 (4-5)	19 (18-21)	19 (18-20)
Perceived health status				
Excellent	25 (25-26)	25 (24-26)	34 (33-36)	34 (33-35)
Very good	31 (30-32)	31 (30-32)	37 (36-39)	38 (37-39)
Good	28 (27-29)	28 (27-29)	22 (21-23)	22 (21-23)
Fair	11 (11-12)	12 (12-13)	5 (5-6)	5 (5-6)
Poor	4 (4-5)	4 (4-4)	1 (1-1)	1 (1-1)
Employed	73 (72-74)	75 (74-76)	88 (87-89)	90 (90-91)
Currently smoke	23 (22-24)	19 (18-20)	11 (10-12)	9 (8-9)
ADL help^c^	2 (2-2)	2 (2-3)	1 (0-1)	1 (1-1)
Instrumental ADL help^c^	4 (3-4)	4 (4-5)	1 (1-2)	1 (1-2)
Annual household income, mean (95% CI), $	23 320 (23 026-23 614)	24 166 (23 750-24 581))	65 047 (63 875-66 219)	67 582 (66 280-68 884)
Family income <100% of FPL	23 (21-24)	22 (21-23)	0	0
Kessler index, mean (95% CI), score^d^	4 (4-4)	3 (3-4)	3 (2-3)	2 (2-2)
Body mass index, mean (95% CI)^e^	28 (28-28)	28 (28-29)	27 (27-27)	28 (27-28)
Chronic diseases, No.^f^				
0	61 (60-62)	62 (61-63)	57 (56-59)	60 (59-61)
1	19 (18-20)	18 (18-19)	22 (22-23)	21 (20-22)
2	9 (8-9)	9 (8-9)	11 (10-11)	11 (10-11)
≥3	11 (10-11)	11 (10-12)	10 (9-10)	8 (8-9)

Abbreviations: ACA, Patient Protection and Affordable Care Act; ADL, activities of daily living; FPL, federal poverty level.

^a^ Percentages are weighted to be nationally representative and to account for nonresponse. Percentages may not sum to 100 because of rounding.

^b^ Includes American Indian, Alaska Native, and any other race or ethnicity not listed.

^c^ A 3-part screener question was used to determine whether the respondent required assistance with ADLs or instrumental ADLs.

^d^ The Kessler index is a measure of nonspecific psychological distress using a sum of 6 psychological distress variables, each on a scale of 0 (none of the time) to 4 (all of the time), with higher scores indicating more distress.

^e^ Body mass index is calculated as weight in kilograms divided by height in meters squared.

^f^ Chronic diseases are among the 20 conditions considered chronic by the Health and Human Services Office of the Assistant Secretary of Health. More details are available in eTable 1 in the Supplement.

We performed all analyses with SAS statistical software version 9.4 (SAS Institute). We considered 2-sided *P* < .05 to be significant. Data analysis was performed from January 2021 to March 2022.

## Results

### Respondent Characteristics

The total sample included 123 171 individuals (mean [SD] age, 39.9 [13.4] years; 65 034 women [52.8%]). Seventy-one percent of US adults aged 18 to 64 years had income below 400% of the FPL. Before implementation of the ACA (2011-2013), those with income less than 400% of the FPL, compared with respondents with income at or above 400% of the FPL, were younger (mean age, 39 [95% CI, 39-39] years vs 44 [95% CI, 43-44] years), more likely to be female (52% [95% CI, 52%-53%] vs 49% [95% CI, 48%-49%]), less likely to be White (56% [95% CI, 53%-58%] vs 75% [95% CI, 73%-77%]), less frequently married or partnered (43% [95% CI, 42%-44%] vs 66% [95% CI, 65%-67%]), more frequently uninsured (26% [95% CI, 25%-27%] vs 7% [95%, 6%-7%]), less frequently employed (73% [95% CI, 72%-74%] vs 88% [95%, 87%-89%]), more frequently smokers (23% [95% CI, 22%-24%] vs 11% [95%, 10%-12%]), and had a similar chronic disease burden; all percentages are weighted (Table 1).

As expected, the uninsurance rate for respondents with income below 400% of the FPL decreased from 26% before the ACA to 18% after the ACA compared with a small decrease from 7% to 5% for those with income over 400% of the FPL (adjusted DiD, −5.61%; 95% CI, −6.89% to −4.34%; *P* < .001). Other differences in sociodemographic characteristics were minimal after implementation of the ACA with the exception of reductions in smoking (23% [95% CI, 22%-24%] of pre-ACA respondents vs 19% [95% CI, 18%-20%] of post-ACA respondents with income <400% of the FPL smoked).

### High-Value Care

In unadjusted and adjusted models, implementation of the ACA was not associated with changes in receipt of high-value care in 4 of 5 composites (Table 2 and eTable 5 in the Supplement). Compared with respondents with income at or above 400% of the FPL, respondents with income less than 400% of the FPL experienced similar changes in receipt of high-value cancer screening, high-value diabetes care, high-value counseling, and high-value medical treatments. In contrast, respondents with income less than 400% of the FPL experienced a small increase in receipt of recommended diagnostic and preventive testing (70% to 72% vs 84% to 84%; adjusted DiD 1.20%; 95% CI, 0.18%-2.21%; *P* = .02).

**Table 2.** Outpatient Quality, Experience, Utilization, and Cost Before (2011-2013) and After (2014-2016) the ACA, by Annual Household Income

Outcome	Patients, mean (95% CI), %				Difference in differences			
	Annual household income <400% FPL		Annual household income ≥400% FPL					
	Before ACA (n = 45 811)	After ACA (n = 41 900)	Before ACA (n = 17 693)	After ACA (n = 17 767)	Unadjusted	*P* value	Adjusted	*P* value
High-value care								
Cancer screening	73 (72-74)	73 (73-74)	80 (79-81)	79 (78-80)	0.64	.48	0.99	.26
Diagnostic and preventive testing	70 (70-71)	72 (72-73)	84 (83-84)	84 (83-84)	2.18^a^	<.001^a^	1.20^a^	.02^a^
Diabetes care	62 (60-63)	59 (58-61)	72 (69-74)	71 (68-74)	−1.34	.58	−0.97	.67
Counseling	45 (44-46)	45 (44-46)	50 (48-51)	49 (48-50)	0.86	.47	−0.55	.59
Medical treatments	34 (33-36)	34 (33-35)	41 (39-43)	39 (37-41)	2.06	.16	0.54	.67
Low-value care								
Antibiotic use	62 (59-64)	56 (52-59)	59 (56-63)	54 (51-57)	−0.76	.82	−4.35	.20
Medical treatments	13 (12-14)	13 (12-14)	8 (7-9)	9 (8-10)	−0.40	.63	−0.99	.28
Imaging	9 (8-10)	10 (9-12)	9 (8-10)	9 (7-11)	1.73	.20	0.77	.60
Respondent experience								
Global rating of health care	69 (68-70)	73 (72-74)	79 (78-81)	81 (80-82)	2.77^a^	.007^a^	2.12^a^	.03^a^
Physician communication	57 (57-58)	63 (62-64)	64 (63-65)	67 (65-68)	2.59^a^	.005^a^	1.86^a^	.04^a^
Access to care	50 (49-51)	54 (53-55)	59 (58-61)	60 (59-62)	3.20^a^	.01^a^	2.58^a^	.047^a^
Utilization								
Encounters, mean (95% CI), No./year								
Office visits	4.3 (4.2-4.5)	4.7 (4.5-4.9)	5.5 (5.3-5.8)	5.8 (5.5-6.1)	0.10	.57	0.12	.50
Emergency department visits	0.2 (0.2-0.2)	0.2 (0.2-0.2)	0.1 (0.1-0.1)	0.1 (0.1-0.1)	−0.01	.27	0.00	.97
Hospital admissions	0.1 (0.1-0.1)	0.1 (0.1-0.1)	0.1 (0.1-0.1)	0.1 (0-0.1)	0.00	.84	0.01	.26
Prescribed medicines, mean (95% CI), total fills/year	10.2 (9.8-10.7)	10.5 (10-11)	9.0 (8.5-9.4)	8.6 (8.2-9)	0.67	.09	0.34	.29
Preventive visit within past year	58 (57-59)	60 (59-61)	69 (68-70)	69 (68-71)	1.81	.07	0.70	.48
Has primary care	65 (64-66)	66 (65-67)	80 (78-81)	77 (76-78)	3.33^a^	<.001^a^	2.97^a^	.001^a^
Cost, $								
Total^b^	3871 (3678-4063)	4309 (4010-4607)	4401 (4120-4683)	4591 (4315-4867)	248.53	.25	195.67	.37
Office-based^b^	867 (824-911)	954 (900-1009)	1206 (1146-1265)	1327 (1248-1406)	−34.36	.52	0.28	>.99
Hospital outpatient department–based^b^	327 (296-357)	411 (345-477)	531 (452-610)	566 (481-652)	49.26	.43	81.31	.20
Inpatient-based^b^	1170 (1069-1270)	1160 (1012-1307)	1061 (876-1246)	933 (769-1097)	118.40	.40	193.15	.21
Prescriptions^b^	944 (830-1057)	1126 (1016-1237)	957 (863-1052)	1045 (930-1161)	94.38	.32	−41.86	.70
Out of pocket								
Total	504 (477-530)	439 (416-461)	757 (719-794)	769 (734-804)	−77.26^a^	.008^a^	−105.50^a^	.001^a^
Office-based	126 (118-134)	131 (119-142)	239 (220-257)	278 (260-296)	−35.05^a^	.006^a^	−45.39^a^	.002^a^
HOD-based	29 (24-35)	23 (20-26)	46 (38-54)	52 (45-59)	−12.20^a^	.04^a^	−14.59^a^	.03^a^
Inpatient-based	49 (36-61)	28 (23-33)	50 (33-67)	38 (31-45)	−8.79	.44	−0.75	.95
Prescriptions	156 (145-167)	127 (116-138)	200 (187-213)	159 (147-171)	12.17	.23	3.14	.76

Abbreviations: ACA, Patient Protection and Affordable Care Act; FPL, federal poverty level.

^a^ Denotes *P* < .05. Regression was adjusted for each year, age, sex, race, ethnicity, US Census region, partner status, education status, health status, employment status, smoking status, activities of daily living, instrumental activities of daily living, Short Form–12 Physical Component Score, Short Form–12 Mental Component Score, body mass index, Kessler index, hypertension, dyslipidemia, diabetes, chronic obstructive pulmonary disease, coronary artery disease or myocardial infarction, cancer, asthma, arthritis, frequency of chronic disease, and primary care. Adjustment did not include primary care for the “has primary care” outcome. See eTable 5 in the Supplement for all measures.

^b^ Includes out-of-pocket expenditures.

### Low-Value Care

Implementation of the ACA was not associated with changes in receipt of low-value care in all 3 composites (Table 2 and eTable 5 in the Supplement). Respondents with income less than 400% of the FPL experienced similar changes in receipt of low-value antibiotic use, low-value medical treatments, and low-value imaging.

### Experience, Communication, and Access

Respondents with income less than 400% of the FPL reported a greater increase in global rating of health care (from 69% to 73% vs from 79% to 81%; adjusted DiD, 2.12%; 95% CI, 0.18%-4.05%; *P* = .03), physician communication (from 57% to 63% vs from 64% to 67%; adjusted DiD, 1.86%; 95% CI, 0.07%-3.64%; *P* = .04), and access to care (from 50% to 54% vs from 59% to 60%; adjusted DiD, 2.58%; 95% CI, 0.05%-5.11%; *P* = .047) compared with respondents with income at or above 400% of the FPL. See Table 2 and eTable 5 in the Supplement for details.

### Utilization and Cost

After implementation of the ACA, we did not observe differences in most measures of utilization, including the proportion of respondents with a preventive visit (change from 58% to 60% vs from 69% to 69%; adjusted DiD, 0.70%; 95% CI, −1.22% to 2.61%; *P* = .48) or filled prescriptions in the past year (change from 10.2 to 10.5 fills vs from 9.0 to 8.6 fills; adjusted DiD, 0.34; 95% CI, −0.29 to 0.98; *P* = .29) (Table 2 and eTable 5 in the Supplement). Respondents with income less than 400% of the FPL mostly maintained receipt of primary care whereas those with income greater than 400% of the FPL generally decreased receipt (change from 65% to 66% vs from 80% to 77%; adjusted DiD, 2.97%; 95% CI, 1.18% to 4.77%; *P* = .001). We also did not observe significant changes in total costs. However, in the post-ACA period, those with income less than 400% of the FPL experienced decreases in total out-of-pocket costs compared with a small increase for higher income individuals (change from $504 to $439 vs from $757 to $769; adjusted DiD, −$105.50; 95% CI, −$167.80 to −$43.20; *P* = .001), which reflected lower out-of-pocket costs for office-based care (change from $126 to $131 vs from $239 to $278; adjusted DiD, −$45.39; 95% CI, −$73.91 to −$16.86; *P* = .002) and hospital outpatient departments (change from $29 to $23 vs from $46 to $52; adjusted DiD, −$14.59; 95% CI, −$27.88 to −$1.29; *P* = .03).

## Discussion

In this nationally representative cross-sectional study, we found that in its first 3 years, the ACA was associated with improved patient experience, communication, and access, and decreased out-of-pocket spending, but with little or no improvement in quality. Surprisingly, the significant improvements in receipt of health insurance and respondent-reported access were not associated with increases in health care utilization or the total cost of care, as has typically been seen when health insurance coverage is extended to more people. Individuals with income less than 400% of the FPL continued to utilize care at lower levels compared with those with higher income.

These findings contrast with a broad series of studies^31,32^ in the health economics literature showing that lowering the cost of obtaining care (eg, as in through providing insurance or lowering copayments) leads to more utilization. In this case, although spending among those with income below 400% of the FPL did increase, it increased no more than for the comparison group with income more than 400% of the FPL. There are several possible explanations for this finding. First, for those at higher income levels, but still below 400% of the FPL, the insurance they accessed as a result of the ACA may come with extremely high deductibles and copayments for some services; these barriers to care might have been sufficient to curtail use in this group, despite out-of-pocket spending decreasing after implementation of the ACA.^33,34^ Second, our findings suggest that low-income patients (many of whom lacked insurance) who were most impacted by the ACA were still using a considerable amount of care before the ACA (although less when compared with the higher income population), whether through community health centers, emergency departments, or subsidized care directly from practitioners. This suggests that a major impact of obtaining insurance coverage in this group is the ability to access more appropriate and/or preferred sites of care and some alleviation of concerns about out-of-pocket payments when doing so, as was found in the Oregon Medicaid experiment.^31^ That we found no increase in use of low-value services also is consistent with this view, as are our findings of improved access and experiences of care. Third, the perception of improved access, without any measurable change in utilization, suggests that obtaining insurance may have lowered individuals’ anxiety regarding their ability to access care.^31^

These findings suggest that insurance reform may be necessary but insufficient to drive changes in the ambulatory quality of care delivered to US individuals. It is possible a larger impact may have been evident had additional states expanded Medicaid. It is also possible that larger changes in out-of-pocket cost may be necessary to impact the receipt of care, or that different cost structures are required to incentivize more high-value care and less low-value care. These findings may suggest that achieving universal insurance coverage could assist in transforming the US health care delivery system into a high-value and equitable system.

Although it was not our primary focus in this study, we note the large and unacceptable chasm in performance on nearly every measure between those with incomes below 400% of the FPL and those with incomes at or above 400% of the FPL (both before and after implementation of the ACA). Other research has shown that these differences hinder the nation’s health care system as a whole and are the result of structural discrimination and racism at all levels of society.^35,36^ Future work should test new policy initiatives to augment the ACA to improve the quality of care received by the most vulnerable individuals, including social care and upstream initiatives.^37^ Our work builds on prior research^3,6,13,38,39,40,41,42^ by extending findings through 2016 with a DiD analysis and comprehensively examining a broad range of measures reflective of the functioning of the health care system including novel analyses of high-value and low-value care, patient experience, communication, access, utilization of pharmacy, preventive visits, and other measures.

### Limitations

Our study has limitations. First, this is an observational study; therefore, we cannot infer causation. Often in DiD analyses the comparison groups are similar except for the policy exposure. Although we adjusted for a rich set of characteristics, unaddressed confounding may still exist. In addition, trends in both comparison groups were similar before ACA implementation. Second, we may have lacked power to detect differences for some measures that had smaller sample sizes. We also did not adjust for multiple comparisons. Third, our quality measures do not address all outpatient care, although to our knowledge, the MEPS represents one of the largest nationally representative sets of consistently collected quality measures. We have previously described the limitations of these measures.^22,43,44,45^ Fourth, our analysis focuses on the entire country. Some states chose not to implement important aspects of the legislation; the MEPS does not make state-based identifiers publicly available, allowing only for a national intention-to-treat analysis, not a state-by-state as-treated analysis. This limited our ability to detect within-state improvements due to the ACA. However, by focusing on the nationwide population with income less than 400% of the FPL, we included in our impacted group all individuals who could benefit from the insurance provisions of the ACA whether it be from subsidized insurance on the exchanges or Medicaid expansion and were able to assess the global association of the policy as it occurred on the ground. Fifth, our data assess only 3 years of the post-ACA experience. However, because of the change in administration in 2016 that included important changes to the ACA and a subsequent increase in the uninsurance rate, analyzing the ACA beyond 2016 would have made for a more challenging appraisal.^20^

## Conclusions

Implementation of the ACA was associated with improved patient experience and access and decreased out-of-pocket expenditures for lower income individuals, but little or no change in quality, utilization, and the total cost of care. Our work suggests that the insurance coverage changes achieved under the ACA are but the first of likely many steps that will be required to improve the US health care system.

## Notes

Supplement. **eTable 1.** Clinical and Patient Experience Quality Measures **eTable 2.** Outpatient Quality, Experience, Utilization, and Cost Before (2011-2013) the Affordable Care Act **eTable 3.** Outpatient Quality, Experience, Utilization, and Cost Before (2011-2013) and After (2016) the Affordable Care Act **eTable 4.** Expanded Measures of Outpatient Quality, Experience, Utilization, and Cost Before (2011-2013) and After (2016) the Affordable Care Act **eTable 5.** Expanded Measures of Outpatient Quality, Experience, Utilization, and Cost Before (2011-2013) and After (2014-2016) the Affordable Care Act **eFigure 1.** High-Value Care Composites, 2011-2016 **eFigure 2.** Low-Value Care Composites, 2011-2016 **eFigure 3.** Experience Composites, 2011-2016 **eFigure 4.** Utilization, 2011-2016 **eFigure 5.** Utilization—Preventive Visit and Primary Care, 2011-2016 **eFigure 6.** Total Costs, 2011-2016 **eFigure 7.** Out-of-Pocket Costs, 2011-2016
