# Supplementary material for: Association of the Patient Protection and Affordable Care Act With Ambulatory Quality, Patient Experience, Utilization, and Cost, 2014-2016
Source: JAMA Netw Open. 2022 Jun 17;5(6):e2218167. doi: 10.1001/jamanetworkopen.2022.18167 (PMC9206183; doi:10.1001/jamanetworkopen.2022.18167)
Supplement: Supplement. — eTable 1. Clinical and Patient Experience Quality Measures eTable 2. Outpatient Quality, Experience, Utilization, and Cost Before (2011-2013) the Affordable Care Act eTable 3. Outpatient Quality, Experience, Utilization, and Cost Before (2011-2013) and After (2016) the Affordable Care Act eTable 4. Expanded Measures of Outpatient Quality, Experience, Utilization, and Cost Before (2011-2013) and After (2016) the Affordable Care Act eTable 5. Expanded Measures of Outpatient Quality, Experience, Utilization, and Cost Before (2011-2013) and After (2014-2016) the Affordable Care Act eFigure 1. High-Value Care Composites, 2011-2016 eFigure 2. Low-Value Care Composites, 2011-2016 eFigure 3. Experience Composites, 2011-2016 eFigure 4. Utilization, 2011-2016 eFigure 5. Utilization—Preventive Visit and Primary Care, 2011-2016 eFigure 6. Total Costs, 2011-2016 eFigure 7. Out-of-Pocket Costs, 2011-2016 [file jamanetwopen-e2218167-s001.pdf]

Supplemental Online Content

Levine DM, Chalasani R, Linder JA, Landon BE. Association of the Patient Protection and Affordable Care Act with ambulatory quality, patient experience, utilization, and cost, 2014-2016. *JAMA Netw Open*. 2022;5(6):e2218167. doi:10.1001/jamanetworkopen.2022.18167

eTable 1. Clinical and Patient Experience Quality Measures
eTable 2. Outpatient Quality, Experience, Utilization, and Cost Before (2011-2013) the Affordable Care Act
eTable 3. Outpatient Quality, Experience, Utilization, and Cost Before (2011-2013) and After (2016) the Affordable Care Act
eTable 4. Expanded Measures of Outpatient Quality, Experience, Utilization, and Cost Before (2011-2013) and After (2016) the Affordable Care Act
eTable 5. Expanded Measures of Outpatient Quality, Experience, Utilization, and Cost Before (2011-2013) and After (2014-2016) the Affordable Care Act
eFigure 1. High-Value Care Composites, 2011-2016
eFigure 2. Low-Value Care Composites, 2011-2016
eFigure 3. Experience Composites, 2011-2016
eFigure 4. Utilization, 2011-2016
eFigure 5. Utilization—Preventive Visit and Primary Care, 2011-2016
eFigure 6. Total Costs, 2011-2016
eFigure 7. Out-of-Pocket Costs, 2011-2016

This supplemental material has been provided by the authors to give readers additional information about their work.

© 2022 Levine DM et al. *JAMA Network Open.*

eTable 1. Clinical and Patient Experience Quality Measures

	Measure	MEPS Data Source[a]	Reference[b]	Numerator	Denominator[c]
Underuse Clinical Care					
	Recommended Cancer Screening				
	Cervical cancer screening	SR	33	Papanicolau smear within past 3 years	Women, age 21-65, no hysterectomy
	Breast cancer screening	SR	34	Mammogram within past 2 years	Women, age 50-74
	Colorectal cancer screening	SR	35	Colonoscopy within past 10 years, sigmoidoscopy within past 5 years, or hemoccult test within past year[d]	Age 50-75
	Recommended Diagnostic and Preventive Testing				
	Dental care	SR	36	Dental visit within 1 year	All
	Blood pressure measurement	SR	37	Blood pressure measurement within 2 years	All
	Cholesterol measurement	SR	38	Cholesterol measurement within any interval	Age >= 35
	Influenza vaccine	SR	39	Influenza vaccine within 1 year	Age >= 50
	Recommended Diabetes Care				
	A1c measurement	DCS	40	A1c measurement at least twice yearly	All with diabetes
	Foot exam	DCS	40	Foot exam within 1 year	All with diabetes
	Eye exam	DCS	40	Retinal exam within 1 year	All with diabetes
	Recommended Counseling				
	Weight loss counseling	SR	41	Within 1 year	All overweight/obese
	Exercise counseling	SR	41	Within 1 year	All overweight/obese
	Smoking cessation counseling	SR	42	Within 1 year	All smokers
	Recommended Medical Treatment				
	Anticoagulation for atrial fibrillation	SR, C, P	43	Anticoagulant prescription	Atrial fibrillation
	ACEi/ARB for heart failure	SR, C, P	44	ACEi/ARB prescription	Heart failure
	Beta blocker for heart failure	SR, C, P	44	Beta blocker prescription	Heart failure
	Salicylates and/or platelet aggregation inhibitors for CAD/MI	SR, C, P	45	Salicylates and/or platelet aggregation inhibitor prescription	CAD/MI
	Beta blocker for CAD/MI	SR, C, P	45	Beta blocker prescription	CAD/MI
	Statin for CAD/MI	SR, C, P	45	Statin prescription	CAD/MI
	Statin for dyslipidemia	SR, C, P		Statin prescription	Dyslipidemia

© 2022 Levine DM et al. *JAMA Network Open.*

Measure	MEPS Data Source[a]	Reference[b]	Numerator	Denominator[c]
ACEi/ARB for diabetes & hypertension	SR, C, P	[40]	ACEi/ARB prescription	Diabetes + hypertension
Statin for CVA	SR, C, P	[46]	Statin prescription	CVA
Antiplatelet for CVA	SR, C, P	[46]	Anti-platelet prescription	CVA
Controller medication for poorly controlled asthma	SR, C, P	[47]	ICS or ICS+LABA	Asthma + systemic steroid in past year
Controller medication for poorly controlled COPD	SR, C, P	[48]	ICS+LABA or LAMA+LABA or ICS+LAMA+LABA	COPD + systemic steroid in past year
Inappropriate Clinical Care				
Inappropriate Antibiotic Use Avoidance				
Antibiotics for acute bronchitis	SR, C, P	[17,50,51]	Antibiotic prescription during visit	Acute bronchitis visit
Antibiotics for acute URI	SR, C, P	[50,51]	Antibiotic prescription during visit	Acute URI visit
Antibiotics for influenza	SR, C, P	[50]	Antibiotic prescription during visit	Influenza visit
Inappropriate Medical Treatment Avoidance				
Benzodiazepine for depression	SR, P	[53]	Benzodiazepine prescription	Depression
Opioid for headache	SR, C, P	[54]	Opioid prescription during visit	Headache visit
Opioid for back pain	SR, C, P	[55]	Opioid prescription during visit	Back pain visit
NSAID use for hypertension, heart failure, or kidney disease	SR, C, P	[56]	NSAID prescription	Hypertension, heart failure, or kidney disease
Inappropriate Imaging Avoidance				
MRI/CT for back pain	C	[57]	MRI/CT within 1 month of visit[e]	Back pain visit
X-ray for back pain	C	[57]	X-ray within 1 month of visit	Back pain visit
MRI/CT for headache	C	[58]	MRI/CT during visit	Headache visit
Patient Experience				
Global Rating of Health Care				
Global rating of health care (0="worst" to 10="best")	SAQ	CAHPS	Top-coded 8, 9, or 10[f]	All
Doctor Communication				
Doctor listened to you (1="never" to 4="always")	SAQ	CAHPS	Top-coded 4[e]	All
Doctor explained so you understood (1-4)	SAQ	CAHPS	Top-coded 4[e]	All
Doctor showed respect (1-4)	SAQ	CAHPS	Top-coded 4[e]	All

© 2022 Levine DM et al. *JAMA Network Open.*

	Measure	MEPS Data Source[a]	Reference[b]	Numerator	Denominator[c]
	Doctor spent enough time with you (1-4)	SAQ	CAHPS	Top-coded 4[e]	All
	Access to Care				
	Got care when ill or injured as soon as wanted (1-4)	SAQ	CAHPS	Top-coded 4[e]	All
	Got medical appointment as soon as wanted (1-4)	SAQ	CAHPS	Top-coded 4[e]	All

[a] MEPS combines multiple data sources (see **Methods** for details): C = clinician; DCS = diabetes care survey; P = pharmacy; SAQ = self-administered questionnaire; SR = self-report of household. Please note that the DCS and SAQ are self-report surveys.

[b] Where possible, these references refer to a detailed discussion of the clinical area that supports each measure. The National Quality Forum and other organizations have adopted these discussions to a measure definition with numerators and denominators (eAppendix for further details). Also, because clinical recommendations change over time, we used consistent measure definitions to ensure valid comparison (see **Methods** for details).

[c] See **eAppendix** for details and sensitivity analyses regarding identification of visit types.

[d] From 2009-2013, we were able to extract the numerator as given. From 2002-2008, we were not able to distinguish between colonoscopy and sigmoidoscopy, nor between a 5-year or 10-year screening interval, nor between screening vs diagnostic procedure. The change in phrasing of this question likely follows the change in recommendations.[59]

[e] Most measures recommend a 6 week delay, but the MEPS only consistently separates visits by month.

[f] "Top-coded" indicates that a response was dichotomized as follows: response of 4 on a 1-4 Likert scale or responses of 8, 9, or 10 on a 0-10 Likert scale were counted as positive. For comparison, we present linear measures rescaled 0 to 10 in the supplement (**eFigure 1**).

Abbreviations: A1c, hemoglobin A1c; ACEi, angiotensin converting enzyme inhibitor; ARB, angiotensin receptor blocker; CAD/MI, coronary artery disease / myocardial infarction; CAHPS, Consumer Assessment of Healthcare Providers and Systems; COPD, chronic obstructive pulmonary disease; CT, computed tomography; CVA, cerebral vascular accident; ICS, inhaled corticosteroid; LABA, long-acting beta agonist; LAMA, long-acting muscarinic antagonist; MRI, magnetic resonance imaging; NSAID, nonsteroidal anti-inflammatory drug; URI, upper respiratory infection

Note: This table was copied from Levine DM, Linder JA, Landon BE. The Quality of Outpatient Care Delivered to Adults in the United States, 2002 to 2013. JAMA Intern Med. 2016;176(12):1778. The references to Figures, Methods, and Appendices refers to the original paper.

© 2022 Levine DM et al. *JAMA Network Open.*

eTable 2. Outpatient Quality, Experience, Utilization, and Cost Before (2011-2013) the Affordable Care Act

	<400% FPL		>=400% FPL		Difference-in-differences	
	2011 (n=14477)	2013 (n=15246)	2011 (n=5609)	2013 (n=5846)	Unadjusted	p
	**Mean % (95% CI)**					
High-Value Care						
Cancer screening	74 (73,75)	72 (71,74)	81 (79,82)	79 (77,80)	0.38	0.80
Diagnostic and preventive testing	71 (70,72)	70 (69,71)	84 (83,85)	83 (82,84)	-0.24	0.76
Diabetes care	63 (60,66)	59 (56,62)	71 (67,75)	73 (69,77)	-5.09	0.17
Counseling	44 (43,45)	45 (44,46)	50 (48,52)	50 (48,52)	0.98	0.56
Medical treatments	35 (33,37)	33 (31,35)	41 (38,43)	41 (39,44)	-2.44	0.27
Low-Value Care						
Antibiotic use	62 (58,67)	59 (54,63)	64 (59,69)	55 (50,61)	4.80	0.33
Medical treatments	12 (11,14)	14 (13,15)	8 (6,9)	8 (7,10)	1.26	0.34
Imaging	9 (7,10)	9 (7,11)	8 (6,10)	10 (7,13)	-2.08	0.39
Respondent Experience						
Global rating of health care	68 (67,70)	70 (69,72)	80 (78,82)	80 (78,82)	2.64	0.15
Physician communication	56 (55,58)	59 (57,60)	64 (62,66)	65 (63,66)	1.41	0.36
Access to care	49 (48,51)	52 (50,54)	59 (57,61)	60 (58,63)	1.93	0.31
Utilization						
Encounters, mean # per year (95% CI)						
Office visits	4.3 (4,4.5)	4.6 (4.4,4.8)	5.5 (5.2,5.9)	5.7 (5.4,6)	0.18	0.58
Emergency department visits	0.2 (0.2,0.2)	0.3 (0.2,0.3)	0.1 (0.1,0.1)	0.1 (0.1,0.1)	**0.04**	**0.004**
Hospital admissions	0.1 (0.1,0.1)	0.1 (0.1,0.1)	0.1 (0.1,0.1)	0.1 (0,0.1)	**0.02**	**0.048**
Prescribed medicines, mean total fills per year (95% CI)	10.5 (9.9,11.1)	10.2 (9.6,10.8)	9.4 (8.8,10)	8.7 (8.1,9.4)	0.30	0.63
Preventive visit within past year	58 (57,60)	57 (56,59)	69 (67,71)	69 (67,70)	-0.63	0.66
Has primary care	67 (65,68)	63 (62,65)	80 (78,81)	80 (78,81)	**-3.21**	**0.02**
Cost						
Total[a]	3787 (3485,4089)	3993 (3727,4259)	4599 (4122,5077)	4219 (3902,4536)	586.45	0.11
Office-based[a]	855 (793,916)	931 (868,994)	1229 (1128,1330)	1218 (1123,1313)	87.58	0.29
HOD-based[a]	352 (300,404)	314 (270,357)	525 (446,604)	479 (368,591)	8.05	0.91
Inpatient-based[a]	1069 (952,1186)	1183 (988,1378)	1220 (850,1590)	885 (698,1072)	448.91	0.08
Prescriptions[a]	992 (749,1235)	944 (834,1054)	964 (848,1080)	959 (809,1108)	-42.49	0.79
Out of pocket, total	515 (480,550)	494 (458,530)	734 (685,782)	789 (725,853)	-75.80	0.11
Out of pocket, office-based	130 (117,143)	129 (117,140)	223 (202,244)	273 (230,317)	-51.08	0.06
Out of pocket, HOD-based	33 (24,43)	27 (19,35)	48 (34,62)	45 (35,56)	-4.15	0.70

© 2022 Levine DM et al. *JAMA Network Open.*

	<400% FPL		>=400% FPL		Difference-in-differences	
	2011 (n=14477)	2013 (n=15246)	2011 (n=5609)	2013 (n=5846)	Unadjusted	p
	**Mean % (95% CI)**					
Out of pocket, inpatient-based	49 (33,65)	45 (27,63)	34 (25,44)	53 (26,81)	-23.13	0.25
Out of pocket, prescriptions	169 (155,182)	141 (125,157)	209 (191,228)	179 (159,198)	2.79	0.88

[a] Includes out-of-pocket expenditures

Note: bold signifies $p < 0.05$.

© 2022 Levine DM et al. *JAMA Network Open.*

eTable 3. Outpatient Quality, Experience, Utilization, and Cost Before (2011-2013) and After (2016) the Affordable Care Act

	**<400% FPL**		**>=400% FPL**		**<400% FPL**			
	Before ACA (n=45811)	After ACA (n=13442)	Before ACA (n=45811)	After ACA (n=13442)	Unadjusted	p	Adjusted	p
	**Mean % (95% CI)**							
High-Value Care								
Cancer screening	73 (72-74)	74 (73-75)	80 (79-81)	80 (78-81)	0.37	0.77	0.70	0.60
Diagnostic and preventive testing	70 (70-71)	73 (72-74)	84 (83-84)	84 (83-85)	**3.06**	**<0.001**	**1.83**	**0.009**
Diabetes care	62 (60-63)	58 (55-61)	72 (69-74)	65 (62-68)	-1.31	0.69	1.18	0.70
Counseling	45 (44-46)	44 (43-46)	50 (48-51)	48 (46-51)	1.04	0.47	-1.09	0.38
Medical treatments	34 (33-36)	34 (32-36)	41 (39-43)	39 (36-42)	1.91	0.34	0.62	0.74
Low-Value Care								
Antibiotic use	62 (59-64)	51 (44-58)	59 (56-63)	49 (42-56)	-0.12	0.98	-3.70	0.56
Medical treatments	13 (12-14)	12 (11-13)	8 (7-9)	9 (8-11)	-2.27	0.06	**-2.94**	**0.03**
Imaging	9 (8-10)	9 (7-11)	9 (8-10)	10 (7-13)	-1.08	0.59	-1.51	0.46
Respondent Experience								
Global rating of health care	69 (68-70)	74 (73-76)	79 (78-81)	80 (79,82)	**4.12**	**0.004**	**2.63**	**0.046**
Physician communication	57 (57-58)	63 (62-65)	64 (63-65)	68 (66,69)	2.25	0.11	0.73	0.60
Access to care	50 (49-51)	56 (54-57)	59 (58-61)	59 (57,62)	**5.65**	**<0.001**	**4.44**	**0.009**
Utilization								
Encounters, mean # per year (95% CI)								
Office visits	4.3 (4.2-4.5)	4.4 (4.2-4.7)	5.5 (5.3-5.8)	5.5 (5.2-5.8)	0.14	0.56	0.07	0.73
Emergency department visits	0.2 (0.2-0.2)	0.2 (0.2-0.2)	0.1 (0.1-0.1)	0.1 (0.1-0.1)	-0.01	0.23	0.00	0.95
Hospital admissions	0.1 (0.1-0.1)	0.1 (0.1-0.1)	0.1 (0.1-0.1)	0 (0-0.1)	0.00	0.63	0.01	0.43
Prescribed medicines, mean total fills per year (95% CI)	10.2 (9.8-10.7)	9.9 (9.4-10.4)	9.0 (8.5-9.4)	8.1 (7.6-8.6)	0.49	0.24	0.13	0.71
Preventive visit within past year	58 (57-59)	61 (60-62)	69 (68-70)	70 (69-72)	1.49	0.24	0.17	0.90
Has primary care	65 (64-66)	66 (65-68)	80 (78-81)	76 (74-77)	**5.08**	**<0.001**	**3.98**	**0.001**
Cost								
Total[a]	3871 (3678-4063)	4313 (3971-4655)	4401 (4120-4683)	4524 (4178-4870)	319.42	0.26	392.11	0.17
Office-based[a]	867 (824-911)	945 (851-1040)	1206 (1146-1265)	1423 (1266-1579)	-138.77	0.15	-21.71	0.81
HOD-based[a]	327 (296-357)	402 (299-505)	531 (452-610)	559 (440-679)	47.64	0.57	47.30	0.56
Inpatient-based[a]	1170 (1069-1270)	1225 (1039-1412)	1061 (876-1246)	843 (676-1009)	274.22	0.09	**420.33**	**0.02**
Prescriptions[a]	944 (830-1057)	1120 (1014-1226)	957 (863-1052)	998 (859-1137)	135.54	0.25	-7.22	0.96
Out of pocket, total	504 (477-530)	428 (397-459)	757 (719-794)	811 (760-862)	**-129.49**	**<0.001**	**-191.02**	**<0.001**

© 2022 Levine DM et al. *JAMA Network Open.*

	<400% FPL		>=400% FPL		<400% FPL			
	Before ACA (n=45811)	After ACA (n=13442)	Before ACA (n=45811)	After ACA (n=13442)	Unadjusted	p	Adjusted	p
Out of pocket, office-based	126 (118-134)	131 (117-144)	239 (220-257)	308 (280-336)	**-64.54**	**<0.001**	**-85.08**	**<0.001**
Out of pocket, HOD-based	29 (24-35)	20 (16-25)	46 (38-54)	52 (42-61)	**-14.71**	**0.03**	-15.42	0.06
Out of pocket, inpatient-based	49 (36-61)	29 (20-38)	50 (33-67)	38 (27-48)	-8.03	0.50	-2.00	0.89
Out of pocket, prescriptions	156 (145-167)	118 (104-132)	200 (187-213)	151 (134-168)	11.08	0.40	-12.96	0.31

[a] Includes out-of-pocket expenditures

Note: bold signifies $p < 0.05$.

Note: regression adjusted for each year, age, gender, race/ethnicity, census region, partner status, education status, health status, employed status, smoking status, ADLs, iADLs, SF-12 PCS, SF-12 MCS, BMI, Kessler, hypertension, dyslipidemia, diabetes, chronic obstructive pulmonary disease, coronary artery disease or myocardial infarction, cancer, asthma, arthritis, frequency of chronic disease, and primary care. Adjustment did not include primary care for the "Has primary care" outcome.

© 2022 Levine DM et al. *JAMA Network Open.*

eTable 4. Expanded Measures of Outpatient Quality, Experience, Utilization, and Cost Before (2011-2013) and After (2016) the Affordable Care Act

	**<400% FPL**				**≥400% FPL**				**Difference in differences**			
	**Before ACA (n=45811)**		**After ACA (n=13442)**		**Before ACA (n=17693)**		**After ACA (n=6212)**					
Measure or Composite	n	Mean (95% CI)	n	Mean (95% CI)	n	Mean (95% CI)			Unadjusted	p	Adjusted	p
High-value cancer screening composite	26027	73 (72-74)	7749	74 (73-75)	10752	80 (79-81)	3721	80 (78-81)	0.37	0.77	0.70	0.60
Cervical cancer screening	19137	85 (84-86)	5554	83 (82-85)	6426	91 (90-92)	2283	90 (89-91)	-0.47	0.67	-0.53	0.65
Breast cancer screening	5886	71 (69-73)	1932	73 (70-75)	3289	84 (82-86)	1130	84 (82-86)	1.83	0.36	2.27	0.27
Colorectal cancer screening	10925	50 (48-51)	3514	55 (52-57)	6661	66 (64-68)	2238	67 (64-70)	3.68	0.08	1.81	0.39
High-value diagnostic and preventive testing composite	45284	70 (70-71)	13223	73 (72-74)	17470	84 (83-84)	6135	84 (83-85)	**3.06**	**<0.001**	**1.83**	**0.009**
Dental checkup	45109	52 (51-53)	13151	56 (54-58)	17399	79 (78-80)	6115	78 (76-79)	**5.16**	**<0.001**	**2.95**	**0.02**
Blood pressure measurement	43961	87 (86-87)	12871	89 (88-90)	17011	93 (93-94)	6006	94 (93-94)	**1.87**	**0.005**	0.74	0.30
Cholesterol measurement	24490	90 (90-91)	7491	92 (91-93)	11996	96 (95-97)	4233	97 (96-97)	0.88	0.26	0.85	0.29
Influenza vaccine	11093	42 (41-44)	3518	47 (45-49)	6725	53 (51-55)	2229	50 (48-53)	**6.93**	**<0.001**	**5.79**	**0.006**
High-value diabetes care composite	3095	62 (60-63)	989	58 (55-61)	932	72 (69-74)	322	65 (62-68)	-1.31	0.69	1.18	0.70
A1c measurement	1975	71 (68-75)	734	71 (67-75)	749	79 (75-83)	274	75 (68-81)	3.80	0.41	7.24	0.14
Foot exam	3043	64 (61-66)	978	59 (55-63)	919	68 (65-72)	321	70 (64-76)	-6.68	0.12	-4.79	0.26
Eye exam	3065	54 (52-57)	978	52 (49-56)	927	70 (66-75)	320	68 (62-74)	0.47	0.92	2.70	0.58

© 2022 Levine DM et al. *JAMA Network Open.*

	<400% FPL				≥400% FPL				Difference in differences			
	**Before ACA (n=45811)**		**After ACA (n=13442)**		**Before ACA (n=17693)**		**After ACA (n=6212)**					
Measure or Composite	n	Mean (95% CI)	n	Mean (95% CI)	n	Mean (95% CI)			Unadjusted	p	Adjusted	p
High-value counseling composite	31762	45 (44-46)	9500	44 (43-46)	11095	50 (48-51)	3965	48 (46-51)	1.04	0.47	-1.09	0.38
Weight loss counseling	29055	39 (37-40)	8827	36 (35-38)	10503	45 (44-47)	3790	43 (40-45)	0.38	0.82	-1.81	0.25
Exercise counseling	29100	46 (45-47)	8862	48 (47-50)	10533	52 (50-53)	3800	53 (50-55)	1.25	0.41	-0.52	0.72
Smoking cessation counseling	7128	60 (59-62)	1786	59 (57-62)	1421	67 (63-70)	415	64 (58-71)	1.39	0.72	-2.02	0.63
High-value medical treatment composite	9298	34 (33-36)	2770	34 (32-36)	4365	41 (39-43)	1416	39 (36-42)	1.91	0.34	0.62	0.74
Anticoagulation for atrial fibrillation	517	19 (14-24)	36	65*	268	19 (13-26)	23	50*	**16.08**	**0.004**	9.90	0.13
ACEi/ARB for heart failure	175	65*	49	68*	29	58*	8	47*	**13.46**	**<0.001**	**21.68**	**<0.001**
Beta blocker for heart failure	175	69*	49	70*	29	82*	8	58*	**25.32**	**<0.001**	**1.70**	**<0.001**
Salicylates and/or platelet aggregation inhibitors for CAD/MI	1410	33 (29-37)	313	33 (27-40)	430	28 (22-34)	90	37 (26-48)	-8.62	0.23	**-18.33**	**<0.001**
Beta blocker for CAD/MI	1410	52 (48-57)	313	55 (48-63)	430	58 (51-65)	90	65 (53-76)	-3.98	0.60	-4.72	0.24
Statin for CAD/MI	1410	52 (48-56)	313	62 (55-68)	430	70 (64-75)	90	73 (60-85)	6.44	0.40	8.22	0.07
Statin for dyslipidemia	5479	64 (62-66)	1660	65 (62-68)	2926	68 (66-71)	956	66 (62-70)	4.33	0.15	3.40	0.27

© 2022 Levine DM et al. *JAMA Network Open.*

	<400% FPL				≥400% FPL				Difference in differences			
	Before ACA (n=45811)		After ACA (n=13442)		Before ACA (n=17693)		After ACA (n=6212)					
Measure or Composite	n	Mean (95% CI)	n	Mean (95% CI)	n	Mean (95% CI)			Unadjusted	p	Adjusted	p
ACEi/ARB for diabetes & hypertension	2465	57 (54-61)	791	65 (60-70)	773	57 (51-62)	262	57 (50-65)	7.12	0.22	7.38	0.24
Statin for CVA	396	46 (37-54)	21	55*	81	50 (36-65)	3	28*	**32.59**	**<0.001**	**21.02**	**<0.001**
Antiplatelet for CVA	396	29 (22-36)	21	8*	81	26 (14-38)	3	47*	**-41.41**	**<0.001**	**-37.68**	**<0.001**
Controller medication for poorly controlled asthma	349	48 (40-55)	115	41*	128	65 (53-76)	57	55*	3.11	0.47	1.62	0.47
Controller medication for poorly controlled COPD	308	24 (18-30)	60	44*	111	24 (14-34)	10	20*	**23.76**	**<0.001**	**17.26**	**<0.001**
Low-value antibiotic use composite	1887	62 (59-64)	419	51 (44-58)	1369	59 (56-63)	316	49 (42-56)	-0.12	0.98	-3.70	0.56
Antibiotics for acute upper respiratory infection	1526	66 (63-69)	263	63 (55-71)	1166	63 (60-67)	225	62 (54-70)	-1.85	0.77	-2.27	0.74
Antibiotics for influenza	375	41 (34-49)	165	25*	224	36 (26-46)	103	16*	**3.64**	**0.02**	-3.34	0.15
Low-value medical treatment composite	12230	13 (12-14)	3696	12 (11-13)	5057	8 (7-9)	1713	9 (8-11)	-2.27	0.06	**-2.94**	**0.03**
Benzodiazepine for depression	3993	11 (9-12)	1357	8 (6-9)	1139	8 (6-10)	430	13 (9-17)	**-8.00**	**0.001**	**-10.22**	**<0.001**
Opioid for headache	575	1 (0-2)	141	2*	244	0 (0-1)	66	1*	0.32	0.67	**-1.06**	**<0.001**
Opioid for back pain	1722	7 (5-8)	500	6 (3-9)	1033	3 (1-4)	351	2 (0-3)	-0.14	0.94	-0.12	0.95

© 2022 Levine DM et al. *JAMA Network Open.*

	<400% FPL				≥400% FPL				Difference in differences			
	Before ACA (n=45811)		After ACA (n=13442)		Before ACA (n=17693)		After ACA (n=6212)					
Measure or Composite	n	Mean (95% CI)	n	Mean (95% CI)	n	Mean (95% CI)			Unadjusted	p	Adjusted	p
NSAID use for hypertension, heart failure, or kidney disease	8395	18 (17-19)	2512	18 (16-20)	3435	11 (10-12)	1129	11 (9-14)	-0.91	0.61	-0.98	0.61
Low-value imaging composite	2251	9 (8-10)	715	9 (7-11)	1252	9 (8-10)	443	10 (7-13)	-1.08	0.59	-1.51	0.46
MRI/CT for back pain	1722	6 (5-7)	500	4 (2-6)	1033	5 (4-7)	351	4 (1-7)	-1.19	0.56	-1.45	0.49
X-ray for back pain	1722	12 (11-14)	500	14 (10-17)	1033	13 (11-15)	351	15 (10-20)	-0.50	0.88	0.20	0.95
MRI/CT for headache	575	7 (4-10)	260	8 (4-12)	244	8 (4-12)	105	11 (4-18)	-1.95	0.69	-3.78	0.33
Respondent experience: global rating of health care	23442	69 (68-70)	6178	74 (73-76)	11386	79 (78-81)	3506	80 (79-82)	**4.12**	**0.004**	**2.63**	**0.046**
Respondent experience: doctor communication composite	23802	57 (57-58)	6284	63 (62-65)	11463	64 (63-65)	3526	68 (66-69)	2.25	0.11	0.73	0.60
Doctor listened to you	23482	59 (58-60)	6169	64 (62-65)	11406	65 (64-67)	3501	67 (65-70)	2.58	0.12	1.20	0.47
Doctor explained so you understood	23694	59 (58-60)	6255	66 (64-67)	11447	66 (65-68)	3523	69 (67-71)	**3.30**	**0.04**	1.79	0.26
Doctor showed respect	23657	63 (61-64)	6247	68 (66-70)	11436	69 (68-70)	3515	73 (71-75)	1.11	0.48	-0.42	0.78
Doctor spent enough time with you	23633	49 (48-50)	6234	56 (54-58)	11430	55 (54-57)	3515	60 (58-62)	2.10	0.22	0.43	0.81

© 2022 Levine DM et al. *JAMA Network Open.*

	<400% FPL				≥400% FPL				Difference in differences			
	**Before ACA (n=45811)**		**After ACA (n=13442)**		**Before ACA (n=17693)**		**After ACA (n=6212)**					
Measure or Composite	n	Mean (95% CI)	n	Mean (95% CI)	n	Mean (95% CI)			Unadjusted	p	Adjusted	p
Respondent experience: access to care composite	23414	50 (49-51)	6057	56 (54-57)	11083	59 (58-61)	3407	59 (57-62)	**5.65**	**<0.001**	**4.44**	**0.009**
Got care when ill or injured as soon as wanted	11189	53 (52-55)	2617	59 (57-62)	3744	68 (66-70)	1108	72 (68-75)	2.58	0.31	0.03	0.99
Got medical appointment as soon as wanted	20448	49 (48-51)	5401	55 (53-57)	10398	57 (56-59)	3217	57 (54-60)	**5.84**	**0.002**	**4.90**	**0.01**
Utilization												
Encounters, mean # per year (95% CI)												
Office visits	45811	4.3 (4.2-4.5)	13442	4.4 (4.2-4.7)	17693	5.5 (5.3-5.8)	6212	5.5 (5.2-5.8)	0.14	0.56	0.07	0.73
Emergency department visits	45811	0.2 (0.2-0.2)	13442	0.2 (0.2-0.2)	17693	0.1 (0.1-0.1)	6212	0.1 (0.1-0.1)	-0.01	0.23	0.00	0.95
Hospital admissions	45811	0.1 (0.1-0.1)	13442	0.1 (0.1-0.1)	17693	0.1 (0.1-0.1)	6212	0 (0-0.1)	0.00	0.63	0.01	0.43
Prescribed medicines, mean total fills per year (95% CI)	45811	10.2 (9.8-10.7)	13442	9.9 (9.4-10.4)	17693	9.0 (8.5-9.4)	6212	8.1 (7.6-8.6)	0.49	0.24	0.13	0.71
Preventive visit within past year	43741	58 (57-59)	12835	61 (60-62)	16935	69 (68-70)	5991	70 (69-72)	1.49	0.24	0.17	0.90
Has primary care	42923	65 (64-66)	12592	66 (65-68)	16527	80 (78-81)	5749	76 (74-77)	**5.08**	**<0.001**	**3.98**	**0.001**
Cost												
Total[c]	45811	3871 (3678-4063)	13442	4313 (3971-4655)	17693	4401 (4120-4683)	6212	4524 (4178-4870)	319.42	0.26	392.11	0.17

© 2022 Levine DM et al. *JAMA Network Open.*

	<400% FPL				≥400% FPL				Difference in differences			
	**Before ACA (n=45811)**		**After ACA (n=13442)**		**Before ACA (n=17693)**		**After ACA (n=6212)**					
Measure or Composite	n	Mean (95% CI)	n	Mean (95% CI)	n	Mean (95% CI)			Unadjusted	p	Adjusted	p
Office-based[c]	45811	867 (824-911)	13442	945 (851-1040)	17693	1206 (1146-1265)	6212	1423 (1266-1579)	-138.77	0.15	-21.71	0.81
HOD-based[c]	45811	327 (296-357)	13442	402 (299-505)	17693	531 (452-610)	6212	559 (440-679)	47.64	0.57	47.30	0.56
Inpatient-based[c]	45811	1170 (1069-1270)	13442	1225 (1039-1412)	17693	1061 (876-1246)	6212	843 (676-1009)	274.22	0.09	**420.33**	**0.02**
Prescriptions[c]	45811	944 (830-1057)	13442	1120 (1014-1226)	17693	957 (863-1052)	6212	998 (859-1137)	135.54	0.25	-7.22	0.96
Out of pocket, total	45811	504 (477-530)	13442	428 (397-459)	17693	757 (719-794)	6212	811 (760-862)	**-129.49**	**<0.001**	**-191.02**	**<0.001**
Out of pocket, office-based	45811	126 (118-134)	13442	131 (117-144)	17693	239 (220-257)	6212	308 (280-336)	**-64.54**	**<0.001**	**-85.08**	**<0.001**
Out of pocket, HOD-based	45811	29 (24-35)	13442	20 (16-25)	17693	46 (38-54)	6212	52 (42-61)	**-14.71**	**0.03**	-15.42	0.06
Out of pocket, inpatient-based	45811	49 (36-61)	13442	29 (20-38)	17693	50 (33-67)	6212	38 (27-48)	-8.03	0.50	-2.00	0.89
Out of pocket, prescriptions	45811	156 (145-167)	13442	118 (104-132)	17693	200 (187-213)	6212	151 (134-168)	11.08	0.40	-12.96	0.31

[a] Includes out-of-pocket expenditures

* Sample size limited such that only one cluster in a stratum for given measure; variance and 95% CI cannot be calculated

Note: bold signifies $p < 0.05$.

Note: regression adjusted for each year, age, gender, race/ethnicity, census region, partner status, education status, health status, employed status, smoking status, ADLs, iADLs, SF-12 PCS, SF-12 MCS, BMI, Kessler, hypertension, dyslipidemia, diabetes, chronic obstructive pulmonary disease, coronary artery disease or myocardial infarction, cancer, asthma, arthritis, frequency of chronic disease, and primary care. Adjustment did not include primary care for the “Has primary care” outcome.

© 2022 Levine DM et al. *JAMA Network Open.*

eTable 5. Expanded Measures of Outpatient Quality, Experience, Utilization, and Cost Before (2011-2013) and After (2014-2016) the Affordable Care Act

	**<400% FPL**				**≥400% FPL**				**Difference in differences**			
	**Before ACA** (n=45811)		**After ACA** (n=41900)		**Before ACA** (n=17693)		**After ACA** (n=17767)					
Measure or Composite	n	Mean (95% CI)	n	Mean (95% CI)	n	Mean (95% CI)	n	Mean (95% CI)	Unadjusted	p	Adjusted	p
High-value cancer screening composite	26027	73 (72-74)	24057	73 (73-74)	10752	80 (79-81)	10698	79 (78-80)	0.64	0.48	0.99	0.26
Cervical cancer screening	19137	85 (84-86)	17459	84 (83-85)	6426	91 (90-92)	6458	90 (90-91)	0.10	0.91	0.29	0.74
Breast cancer screening	5886	71 (69-73)	5869	72 (70-74)	3289	84 (82-86)	3261	83 (82-85)	1.83	0.26	2.41	0.14
Colorectal cancer screening	10925	50 (48-51)	10671	52 (51-54)	6661	66 (64-68)	6576	66 (64-68)	2.46	0.13	1.22	0.45
High-value diagnostic and preventive testing composite	45284	70 (70-71)	41299	72 (72-73)	17470	84 (83-84)	17562	84 (83-84)	**2.18**	**<0.001**	**1.20**	**0.02**
Dental checkup	45109	52 (51-53)	41069	54 (53-56)	17399	79 (78-80)	17499	78 (76-79)	**3.58**	**<0.001**	**2.23**	**0.02**
Blood pressure measurement	43961	87 (86-87)	40279	88 (88-89)	17011	93 (93-94)	17189	94 (93-94)	**1.52**	**0.003**	0.60	0.25
Cholesterol measurement	24490	90 (90-91)	23073	92 (91-92)	11996	96 (95-97)	12053	97 (96-97)	0.99	0.10	1.02	0.11
Influenza vaccine	11093	42 (41-44)	10751	45 (43-46)	6725	53 (51-55)	6577	52 (50-54)	**3.46**	**0.03**	1.85	0.25
High-value diabetes care composite	3095	62 (60-63)	2995	59 (58-61)	932	72 (69-74)	856	71 (68-74)	-1.34	0.58	-0.97	0.67
A1c measurement	1975	71 (68-75)	2138	70 (68-73)	749	79 (75-83)	704	77 (73-81)	1.07	0.77	2.61	0.47
Foot exam	3043	64 (61-66)	2959	62 (60-64)	919	68 (65-72)	848	71 (67-75)	**9.02**	**<0.001**	-4.07	0.16
Eye exam	3065	54 (52-57)	2967	52 (49-55)	927	70 (66-75)	847	68 (64-72)	-0.14	0.97	-0.58	0.87

© 2022 Levine DM et al. *JAMA Network Open.*

	<400% FPL				≥400% FPL				Difference in differences			
	**Before ACA** (n=45811)		**After ACA** (n=41900)		**Before ACA** (n=17693)		**After ACA** (n=17767)					
Measure or Composite	n	Mean (95% CI)	n	Mean (95% CI)	n	Mean (95% CI)	n	Mean (95% CI)	Unadjusted	p	Adjusted	p
High-value counseling composite	31762	45 (44-46)	29395	45 (44-46)	11095	50 (48-51)	11233	49 (48-50)	0.86	0.47	-0.55	0.59
Weight loss counseling	29055	39 (37-40)	27237	37 (36-38)	10503	45 (44-47)	10765	44 (42-45)	0.29	0.82	-1.02	0.39
Exercise counseling	29100	46 (45-47)	27291	48 (47-49)	10533	52 (50-53)	10793	53 (51-55)	0.71	0.59	-0.61	0.61
Smoking cessation counseling	7128	60 (59-62)	5694	61 (59-63)	1421	67 (63-70)	1179	64 (60-68)	2.73	0.34	-0.11	0.97
High-value medical treatment composite	9298	34 (33-36)	8875	34 (33-35)	4365	41 (39-43)	4179	39 (37-41)	2.06	0.16	0.54	0.67
Anticoagulation for atrial fibrillation	517	19 (14-24)	416	31 (23-39)	268	19 (13-26)	196	23 (15-31)	**8.78**	**<0.001**	2.80	0.32
ACEi/ARB for heart failure	175	65*	172	63*	29	58*	23	67*				
Beta blocker for heart failure	175	69*	172	67*	29	82*	23	77*				
Salicylates and/or platelet aggregation inhibitors for CAD/MI	1410	33 (29-37)	1229	33 (29-37)	430	28 (22-34)	349	30 (23-36)	-1.80	0.71	-8.67	0.07
Beta blocker for CAD/MI	1410	52 (48-57)	1229	53 (49-58)	430	58 (51-65)	349	51 (44-58)	7.68	0.19	4.98	0.40
Statin for CAD/MI	1410	52 (48-56)	1229	56 (52-60)	430	70 (64-75)	349	63 (55-71)	**10.69**	**0.049**	4.44	0.29
Statin for dyslipidemia	5479	64 (62-66)	5206	64 (62-67)	2926	68 (66-71)	2681	67 (65-70)	1.66	0.45	1.55	0.48

© 2022 Levine DM et al. *JAMA Network Open.*

	<400% FPL				≥400% FPL				Difference in differences			
	**Before ACA** (n=45811)		**After ACA** (n=41900)		**Before ACA** (n=17693)		**After ACA** (n=17767)					
Measure or Composite	n	Mean (95% CI)	n	Mean (95% CI)	n	Mean (95% CI)	n	Mean (95% CI)	Unadjusted	p	Adjusted	p
ACEi/ARB for diabetes & hypertension	2465	57 (54-61)	2518	63 (60-66)	773	57 (51-62)	734	61 (56-66)	1.53	0.74	2.15	0.68
Statin for CVA	396	46 (37-54)	300	57 (49-65)	81	50 (36-65)	64	49 (33-65)	**12.68**	**0.03**	-2.63	0.42
Antiplatelet for CVA	396	29 (22-36)	300	18 (12-25)	81	26 (14-38)	64	41 (25-57)	**-25.55**	**<0.001**	**-24.12**	**<0.001**
Controller medication for poorly controlled asthma	349	48 (40-55)	405	47 (40-53)	128	65 (53-76)	177	58 (48-67)	**6.06**	**0.13**	**8.31**	**0.009**
Controller medication for poorly controlled COPD	308	24 (18-30)	304	36 (28-43)	111	24 (14-34)	97	30 (19-40)	**5.94**	**<0.001**	0.00	1.00
Low-value antibiotic use composite	1887	62 (59-64)	1673	56 (52-59)	1369	59 (56-63)	1284	54 (51-57)	-0.76	0.82	-4.35	0.20
Antibiotics for acute upper respiratory infection	1526	66 (63-69)	1283	63 (60-67)	1166	63 (60-67)	1014	61 (57-65)	-0.18	0.96	-3.43	0.31
Antibiotics for influenza	375	41 (34-49)	428	27 (21-33)	224	36 (26-46)	300	28 (21-35)	-6.10	0.07	**-9.09**	**0.04**
Low-value medical treatment composite	12230	13 (12-14)	11567	13 (12-14)	5057	8 (7-9)	5107	9 (8-10)	-0.40	0.63	-0.99	0.28
Benzodiazepine for depression	3993	11 (9-12)	4094	8 (7-10)	1139	8 (6-10)	1239	9 (7-12)	-3.40	0.05	-3.68	0.07
Opioid for headache	575	1 (0-2)	512	3 (1-6)	244	0 (0-1)	224	1 (-1-4)	1.06	0.56	-0.96	<0.01
Opioid for back pain	1722	7 (5-8)	1802	9 (7-11)	1033	3 (1-4)	1179	3 (2-5)	1.51	0.32	0.54	0.74

© 2022 Levine DM et al. *JAMA Network Open.*

	<400% FPL				≥400% FPL				Difference in differences			
	**Before ACA** (n=45811)		**After ACA** (n=41900)		**Before ACA** (n=17693)		**After ACA** (n=17767)					
Measure or Composite	n	Mean (95% CI)	n	Mean (95% CI)	n	Mean (95% CI)	n	Mean (95% CI)	Unadjusted	p	Adjusted	p
NSAID use for hypertension, heart failure, or kidney disease	8395	18 (17-19)	7812	19 (18-21)	3435	11 (10-12)	3297	12 (11-14)	0.36	0.76	0.05	0.97
Low-value imaging composite	2251	9 (8-10)	2333	10 (9-12)	1252	9 (8-10)	1397	9 (7-11)	1.73	0.20	0.77	0.60
MRI/CT for back pain	1722	6 (5-7)	1802	7 (5-9)	1033	5 (4-7)	1179	5 (3-6)	1.46	0.35	1.22	0.48
X-ray for back pain	1722	12 (11-14)	1802	15 (13-17)	1033	13 (11-15)	1179	13 (11-16)	3.04	0.16	2.46	0.33
MRI/CT for headache	575	7 (4-10)	631	9 (6-12)	244	8 (4-12)	263	9 (5-14)	0.33	0.92	-1.80	0.43
Respondent experience: global rating of health care	23442	69 (68-70)	19259	73 (72-74)	11386	79 (78-81)	10293	81 (80-82)	**2.77**	**0.007**	**2.12**	**0.03**
Respondent experience: doctor communication composite	23802	57 (57-58)	19572	63 (62-64)	11463	64 (63-65)	10362	67 (65-68)	**2.59**	**0.005**	**1.86**	**0.04**
Doctor listened to you	23482	59 (58-60)	19273	63 (62-64)	11406	65 (64-67)	10295	67 (66-68)	**2.63**	**0.01**	2.03	0.05
Doctor explained so you understood	23694	59 (58-60)	19498	65 (64-66)	11447	66 (65-68)	10353	69 (67-70)	**3.07**	**0.003**	**2.32**	**0.02**
Doctor showed respect	23657	63 (61-64)	19470	68 (66-69)	11436	69 (68-70)	10335	72 (71-73)	2.01	0.05	1.32	0.20
Doctor spent enough time with you	23633	49 (48-50)	19416	55 (54-56)	11430	55 (54-57)	10334	58 (57-60)	**2.67**	**0.02**	1.79	0.13

© 2022 Levine DM et al. *JAMA Network Open.*

	<400% FPL				≥400% FPL				Difference in differences			
	**Before ACA** (n=45811)		**After ACA** (n=41900)		**Before ACA** (n=17693)		**After ACA** (n=17767)					
Measure or Composite	n	Mean (95% CI)	n	Mean (95% CI)	n	Mean (95% CI)	n	Mean (95% CI)	Unadjusted	p	Adjusted	p
Respondent experience: access to care composite	23414	50 (49-51)	19222	54 (53-55)	11083	59 (58-61)	10065	60 (59-62)	**3.20**	**0.01**	**2.58**	**0.047**
Got care when ill or injured as soon as wanted	11189	53 (52-55)	8495	57 (55-59)	3744	68 (66-70)	3295	71 (69-73)	0.77	0.68	-0.31	0.87
Got medical appointment as soon as wanted	20448	49 (48-51)	16994	53 (52-54)	10398	57 (56-59)	9466	58 (56-60)	**3.36**	**0.02**	**2.83**	**0.048**
Utilization												
Encounters, mean # per year (95% CI)												
Office visits	45811	4.3 (4.2-4.5)	41900	4.7 (4.5-4.9)	17693	5.5 (5.3-5.8)	17767	5.8 (5.5-6.1)	0.10	0.57	0.12	0.50
Emergency department visits	45811	0.2 (0.2-0.2)	41900	0.2 (0.2-0.2)	17693	0.1 (0.1-0.1)	17767	0.1 (0.1-0.1)	-0.01	0.27	0.00	0.97
Hospital admissions	45811	0.1 (0.1-0.1)	41900	0.1 (0.1-0.1)	17693	0.1 (0.1-0.1)	17767	0.1 (0-0.1)	0.00	0.84	0.01	0.26
Prescribed medicines, mean total fills per year (95% CI)	45811	10.2 (9.8-10.7)	41900	10.5 (10-11)	17693	9.0 (8.5-9.4)	17767	8.6 (8.2-9)	0.67	0.09	0.34	0.29
Preventive visit within past year	43741	58 (57-59)	40117	60 (59-61)	16935	69 (68-70)	17158	69 (68-71)	1.81	0.07	0.70	0.48
Has primary care	42923	65 (64-66)	39182	66 (65-67)	16527	80 (78-81)	16518	77 (76-78)	**3.33**	**<0.001**	**2.97**	**0.001**

© 2022 Levine DM et al. *JAMA Network Open.*

	<400% FPL				≥400% FPL				Difference in differences			
	**Before ACA** (n=45811)		**After ACA** (n=41900)		**Before ACA** (n=17693)		**After ACA** (n=17767)					
Measure or Composite	n	Mean (95% CI)	n	Mean (95% CI)	n	Mean (95% CI)	n	Mean (95% CI)	Unadjusted	p	Adjusted	p
Cost												
Total[c]	45811	3871 (3678-4063)	41900	4309 (4010-4607)	17693	4401 (4120-4683)	17767	4591 (4315-4867)	248.53	0.25	195.67	0.37
Office-based[c]	45811	867 (824-911)	41900	954 (900-1009)	17693	1206 (1146-1265)	17767	1327 (1248-1406)	-34.36	0.52	0.28	1.00
HOD-based[c]	45811	327 (296-357)	41900	411 (345-477)	17693	531 (452-610)	17767	566 (481-652)	49.26	0.43	81.31	0.20
Inpatient-based[c]	45811	1170 (1069-1270)	41900	1160 (1012-1307)	17693	1061 (876-1246)	17767	933 (769-1097)	118.40	0.40	193.15	0.21
Prescriptions[c]	45811	944 (830-1057)	41900	1126 (1016-1237)	17693	957 (863-1052)	17767	1045 (930-1161)	94.38	0.32	-41.86	0.70
Out of pocket, total	45811	504 (477-530)	41900	439 (416-461)	17693	757 (719-794)	17767	769 (734-804)	**-77.26**	**0.008**	**-105.50**	**0.001**
Out of pocket, office-based	45811	126 (118-134)	41900	131 (119-142)	17693	239 (220-257)	17767	278 (260-296)	**-35.05**	**0.006**	**-45.39**	**0.002**
Out of pocket, HOD-based	45811	29 (24-35)	41900	23 (20-26)	17693	46 (38-54)	17767	52 (45-59)	**-12.20**	**0.04**	**-14.59**	**0.03**
Out of pocket, inpatient-based	45811	49 (36-61)	41900	28 (23-33)	17693	50 (33-67)	17767	38 (31-45)	-8.79	0.44	-0.75	0.95
Out of pocket, prescriptions	45811	156 (145-167)	41900	127 (116-138)	17693	200 (187-213)	17767	159 (147-171)	12.17	0.23	3.14	0.76

[a] Includes out-of-pocket expenditures
* Sample size limited such that only one cluster in a stratum for given measure; variance and 95% CI cannot be calculated
Note: bold signifies p < 0.05.

© 2022 Levine DM et al. *JAMA Network Open.*

Note: regression adjusted for each year, age, gender, race/ethnicity, census region, partner status, education status, health status, employed status, smoking status, ADLs, iADLs, SF-12 PCS, SF-12 MCS, BMI, Kessler, hypertension, dyslipidemia, diabetes, chronic obstructive pulmonary disease, coronary artery disease or myocardial infarction, cancer, asthma, arthritis, frequency of chronic disease, and primary care. Adjustment did not include primary care for the "Has primary care" outcome.

© 2022 Levine DM et al. *JAMA Network Open.*

eFigure 1: High-Value Care Composites, 2011-2016

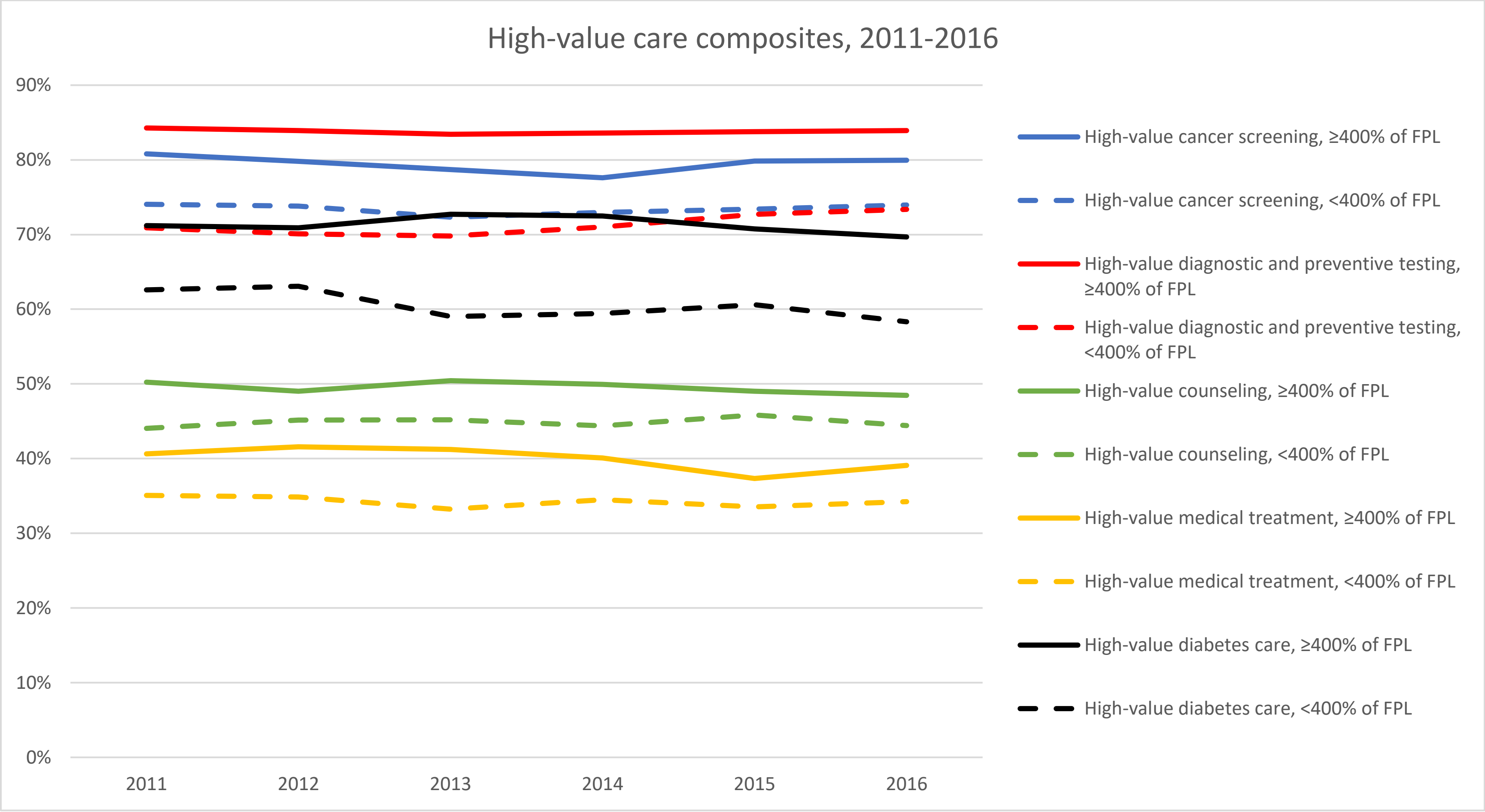

© 2022 Levine DM et al. *JAMA Network Open.*

eFigure 2: Low-Value Care Composites, 2011-2016

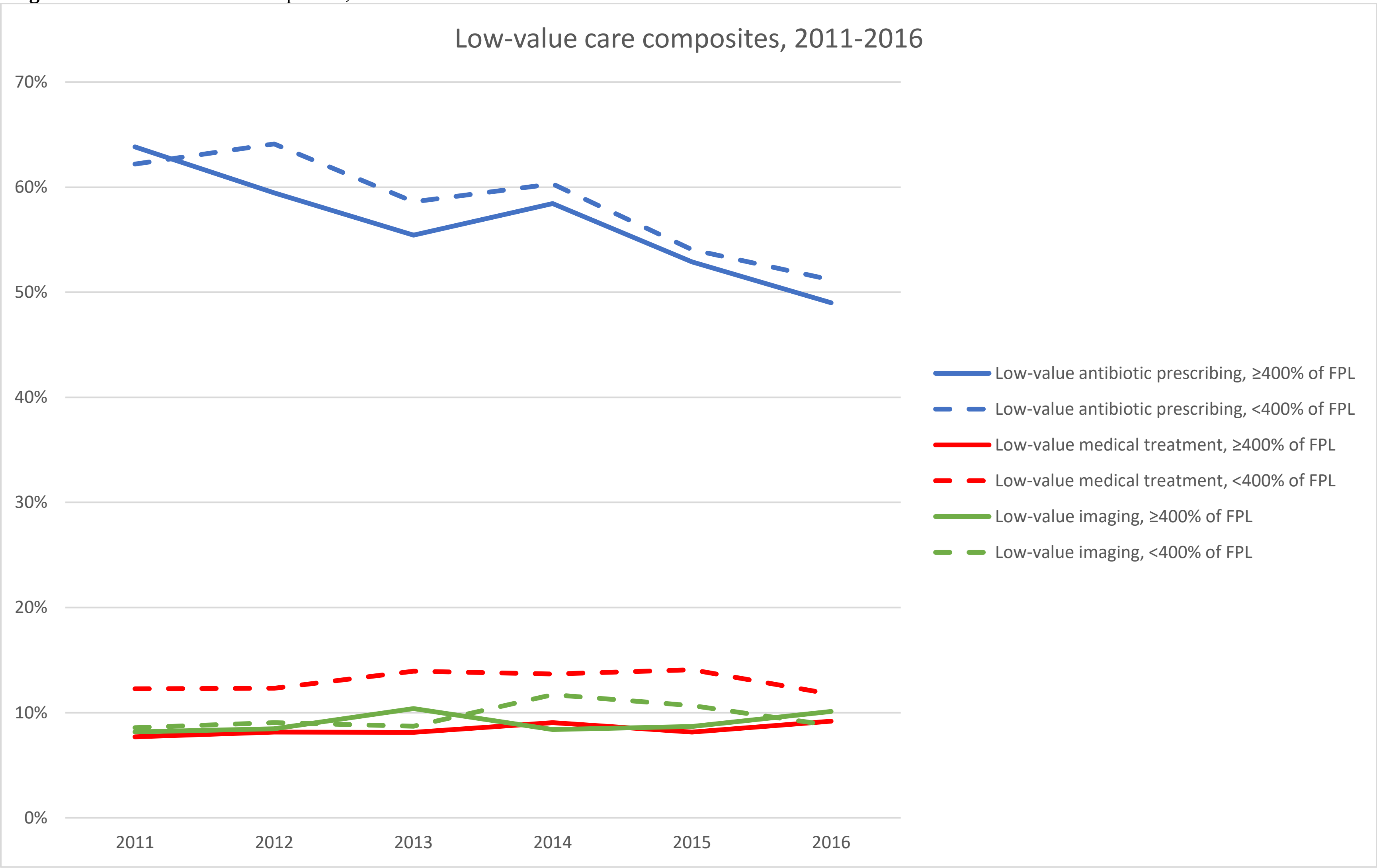

© 2022 Levine DM et al. *JAMA Network Open.*

eFigure 3: Experience Composites, 2011-2016

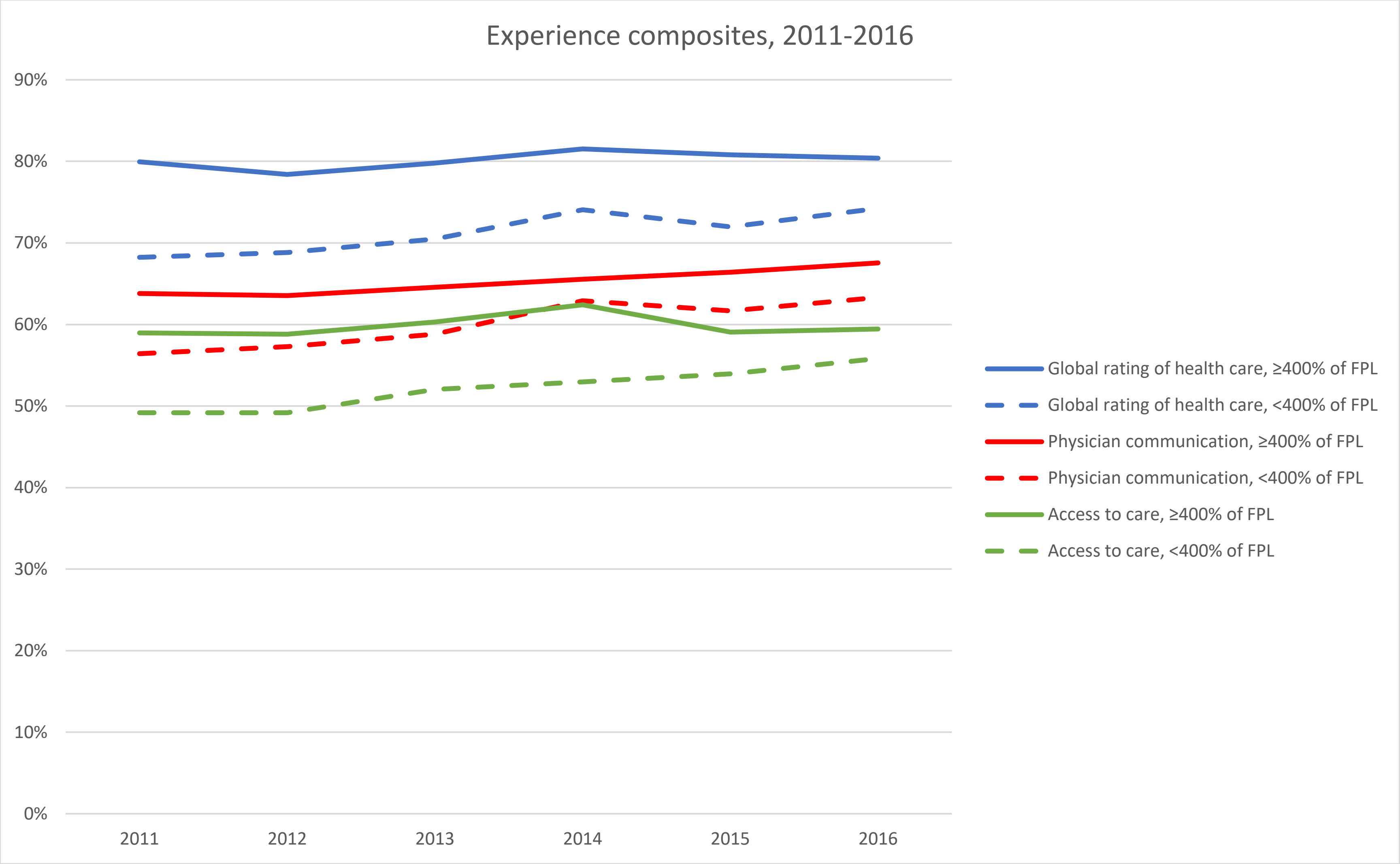

© 2022 Levine DM et al. *JAMA Network Open.*

eFigure 4: Utilization, 2011-2016

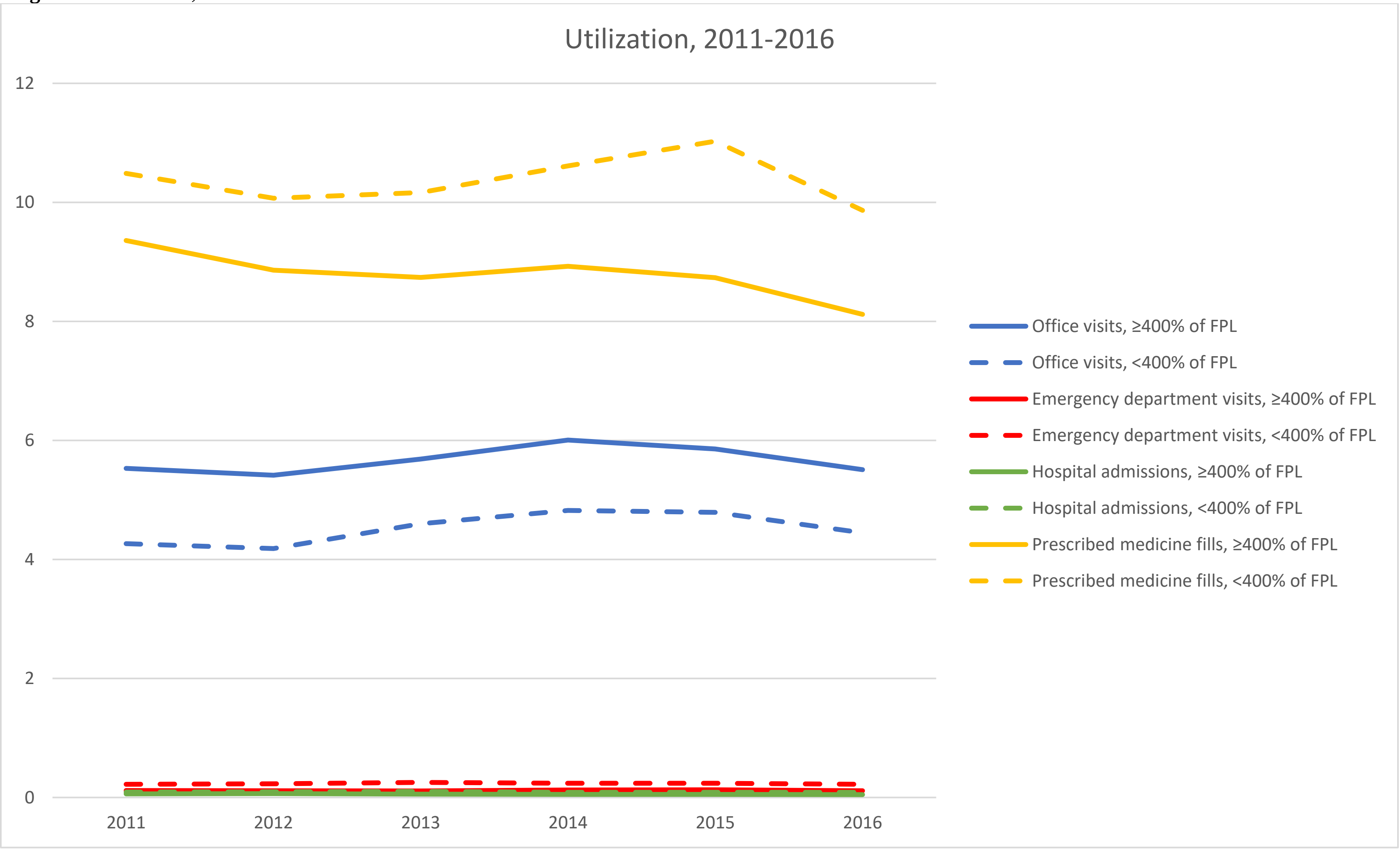

© 2022 Levine DM et al. *JAMA Network Open.*

eFigure 5: Utilization—Preventive Visit and Primary Care, 2011-2016

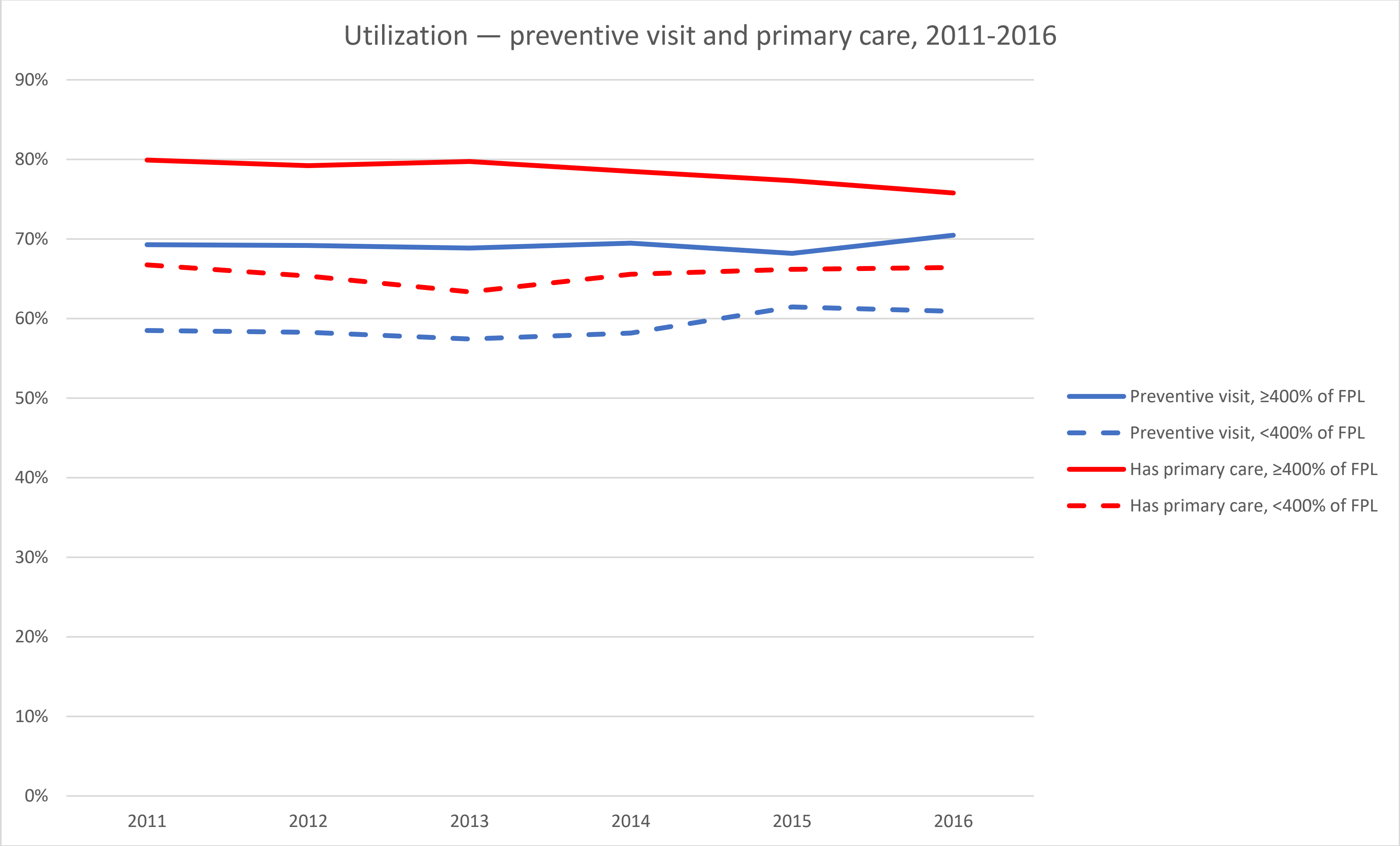

© 2022 Levine DM et al. *JAMA Network Open.*

eFigure 6: Total Costs, 2011-2016

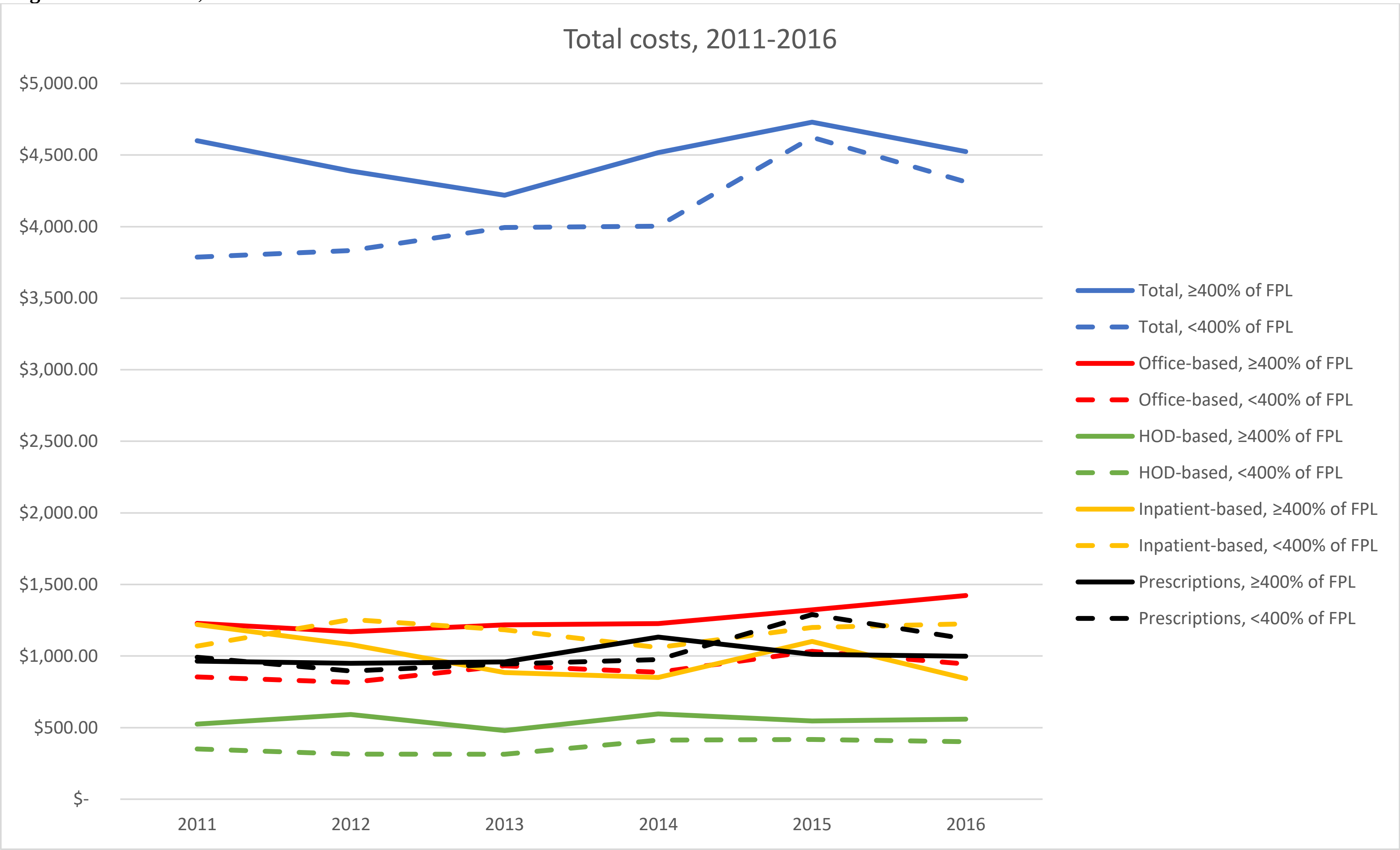

© 2022 Levine DM et al. *JAMA Network Open.*

eFigure 7: Out-of-Pocket Costs, 2011-2016

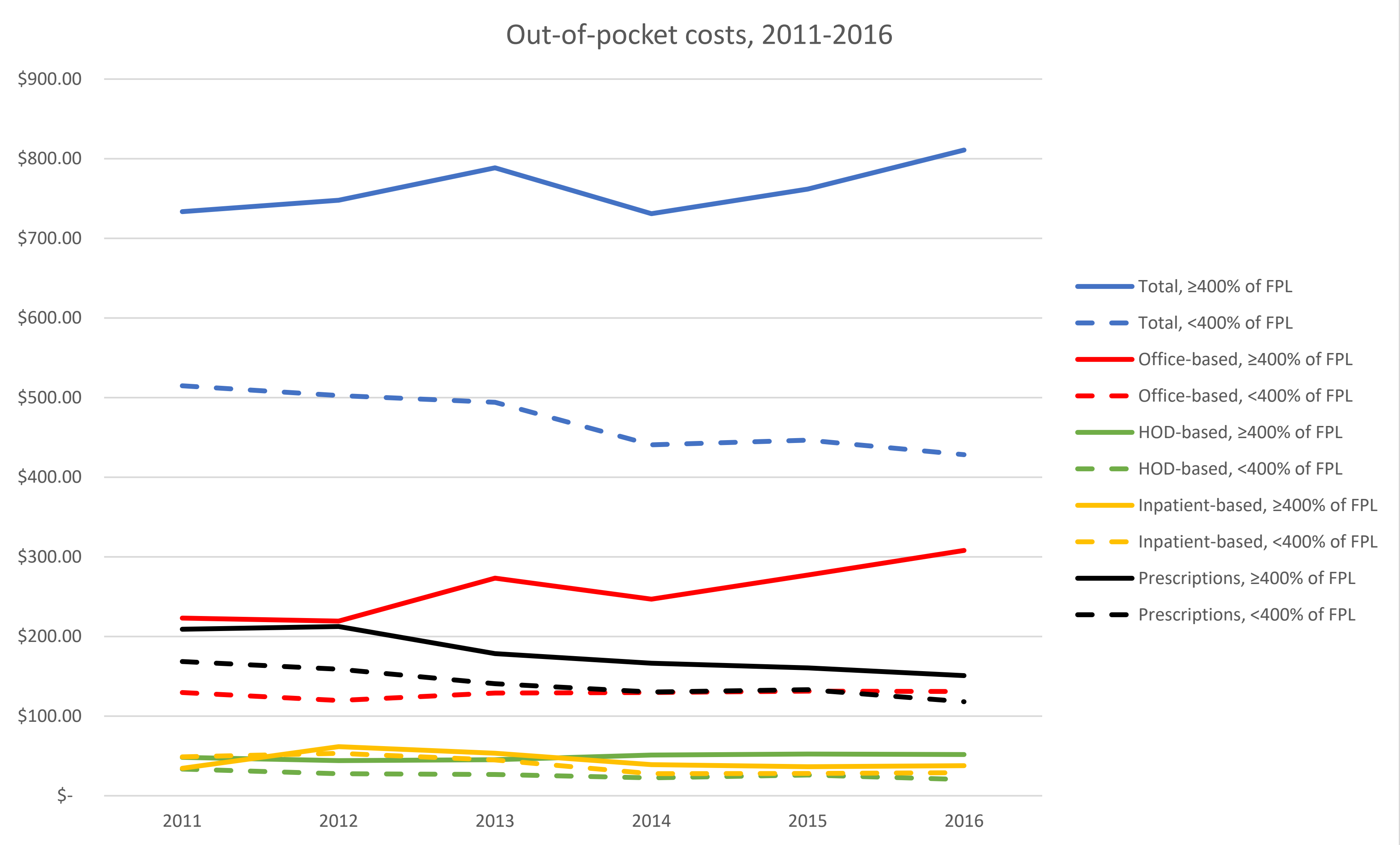

© 2022 Levine DM et al. *JAMA Network Open.*
